# Acyl-CoA oxidase ACOX-1 interacts with a peroxin PEX-5 to play roles in larval development of *Haemonchus contortus*

**DOI:** 10.1371/journal.ppat.1009767

**Published:** 2021-07-16

**Authors:** Hengzhi Shi, Xiaocui Huang, Xueqiu Chen, Yi Yang, Zhao Wang, Yimin Yang, Fei Wu, Jingru Zhou, Chaoqun Yao, Guangxu Ma, Aifang Du

**Affiliations:** 1 Institute of Preventive Veterinary Medicine, Zhejiang Provincial Key Laboratory of Preventive Veterinary Medicine, College of Animal Sciences, Zhejiang University, Hangzhou, Zhejiang, China; 2 Shanghai Institute of Materia Medica, Chinese Academy of Science, Shanghai, China; 3 Department of Biomedical Sciences and One Health Center for Zoonoses and Tropical Veterinary Medicine, Ross University School of Veterinary Medicine, Basseterre, St. Kitts & Nevis; 4 Department of Veterinary Biosciences, Melbourne Veterinary School, University of Melbourne, Parkville, Victoria, Australia; University of Cambridge, UNITED KINGDOM

## Abstract

Hypobiosis (facultative developmental arrest) is the most important life-cycle adaptation ensuring survival of parasitic nematodes under adverse conditions. Little is known about such survival mechanisms, although ascarosides (ascarylose with fatty acid-derived side chains) have been reported to mediate the formation of dauer larvae in the free-living nematode *Caenorhabditis elegans*. Here, we investigated the role of a key gene *acox-1*, in the larval development of *Haemonchus contortus*, one of the most important parasitic nematodes that employ hypobiosis as a routine survival mechanism. In this parasite, *acox-1* encodes three proteins (ACOXs) that all show a fatty acid oxidation activity *in vitro* and *in vivo*, and interact with a peroxin PEX-5 in peroxisomes. In particular, a peroxisomal targeting signal type1 (PTS1) sequence is required for ACOX-1 to be recognised by PEX-5. Analyses on developmental transcription and tissue expression show that *acox-1* is predominantly expressed in the intestine and hypodermis of *H*. *contortus*, particularly in the early larval stages in the environment and the arrested fourth larval stage within host animals. Knockdown of *acox-1* and *pex-5* in parasitic *H*. *contortus* shows that these genes play essential roles in the post-embryonic larval development and likely in the facultative arrest of this species. A comprehensive understanding of these genes and the associated β-oxidation cycle of fatty acids should provide novel insights into the developmental regulation of parasitic nematodes, and into the discovery of novel interventions for species of socioeconomic importance.

## Author summary

*Haemonchus contortus* is one of the most pathogenic nematodes causing substantial economic losses worldwide that undergo facultative arrest (diapause) to survive harsh environmental and host conditions. However, the molecular basis (e.g., fatty acid oxidation and dauer pheromone synthesis) underlying such survival mechanisms has not yet been elucidated in parasitic nematodes. Here, we show that an acyl-CoA oxidase ACOX-1 interacts with a peroxin PEX-5 and functions in fatty acid oxidation in peroxisomes. Further, knockdown of ACOX-1 and PEX-5 results in a lethal phenotype of the infective stage and likely developmental parasitic larval stage within host animals. This data demonstrates an essential role of ACOX-1-PEX-5 and potentially ascarosides signalling in the facultative arrested larval development of parasitic nematodes, and suggests possible intervention targets.

## Introduction

Temporary cessation of development among nematodes in response to certain circumstances or within certain host animals, known as facultative developmental arrest or hypobiosis, is an ability to interrupt the life cycle to survive harsh conditions [1–3]. For instance, in the free-living nematode *Caenorhabditis elegans*, larvae arrest their development at the third-larval stage (L3) and enter a stress-resistant dauer stage under adverse conditions such as food shortage, high population density, or high temperature [4,5]. Similarly, in parasitic nematodes (e.g., Ancylostomatidae, Ascaridae, Strongyloididae, Trichostrongylidae, Trichonematidae), infective larvae can also cease their development at an early parasitic stage in response to seasonal/host factors, and do not resume their development until conditions become favourable [1,6,7]. Mechanisms underlying such developmental arrest of nematodes and related phenomena have been proposed and investigated for decades [1–3], with advanced understanding achieved mostly in the model organism *C*. *elegans* [8–13].

Dauer pheromones play crucial roles in the developmental arrest of *C*. *elegans*, representing a class of 3, 6-dideoxy-L-sugar ascarylose linked with a fatty acid-derived side chain (ascarosides) [14,15]. At least five ascarosides, such as asc-C6-MK (C6; ascr#2), asc-ΔC9 (C9; ascr#3), asc-ωC3 (C3; ascr#5), asc-ΔC7-PABA (ascr#8) and IC-asc-C5 (C5; icas#9), serve as dauer pheromones and play roles in developmental regulation of *C*. *elegans* [15–17]. These molecules are sensed by G protein-coupled receptors (GPCRs) of specific chemosensory neurons [16,18–20], inhibiting cyclic guanosine monophosphate (cGMP) [21,22], transforming growth factor β (TGF-β) [23,24], and insulin/insulin-like growth factor 1 (IGF-1) [25–27], which converge on steroid hormone receptor inactivation for a molecular decision of dauer formation [28,29]. Apart from *C*. *elegans*, ascarosides have also been detected in a range of free-living and parasitic nematodes, suggesting conserved ascarosides synthesis and signalling machinery among nematodes [30–32]. However, the genetic basis for such machinery has not yet been elucidated in parasitic nematodes, particularly species of veterinary and medical importance.

The peroxisomal fatty acid β-oxidation cycle functions in long-chain fatty acid degradation, and in biosynthesis of the short-chain ascarosides in *C*. *elegans* [10,11,33,34]. This β-oxidation cycle involves an acyl-CoA oxidase (ACOX-1), an enoyl-CoA hydratase (MAOC-1), a (3*R*)-hydroxyacyl-CoA dehydrogenase (DHS-28) and a 3-ketoacyl-CoA thiolase (DAF-22) that function in the oxidation, hydration, dehydration and thiolysis of fatty acids, respectively [35,36]. In particular, mutation of these molecules leads to compromised production of short-chain ascarosides in *C*. *elegans* [10,11], suggesting their important roles in dauer pheromone synthesis. Nonetheless, little is known about this fatty acid β-oxidation pathway at the molecular level in major parasitic nematodes of socioeconomic importance.

In our previous work [37–40], three orthologues of genes *maoc-1*, *dhs-28* and *daf-22* involved in the fatty acid β-oxidation pathway have been identified in *H*. *contortus* (Trichostrongylidae; the barber’s pole worm), one of the most pathogenic worms that enter developmental arrest in the infective juvenile stage and diapause of the early fourth larval stage (L4) within small ruminants [41–43]. Here, we identified and functionally characterised the previously unknown gene *acox-1* in the fatty acid β-oxidation pathway of this parasitic nematode of global economic importance. Fatty acid oxidation activities of *acox-1* encoded proteins tested *in vitro* and *in vivo*. Subcellular localisation, tissue expression, developmental transcription and RNA interference (RNAi)-mediated gene knockdown analyses were conducted to investigate the functions of ACOX-1 in the fatty acid β-oxidation and developmental arrest of the parasitic nematode *H*. *contortus*.

## Results

### *Hc-acox-1* encodes three transcript variants

**Fig 1:**
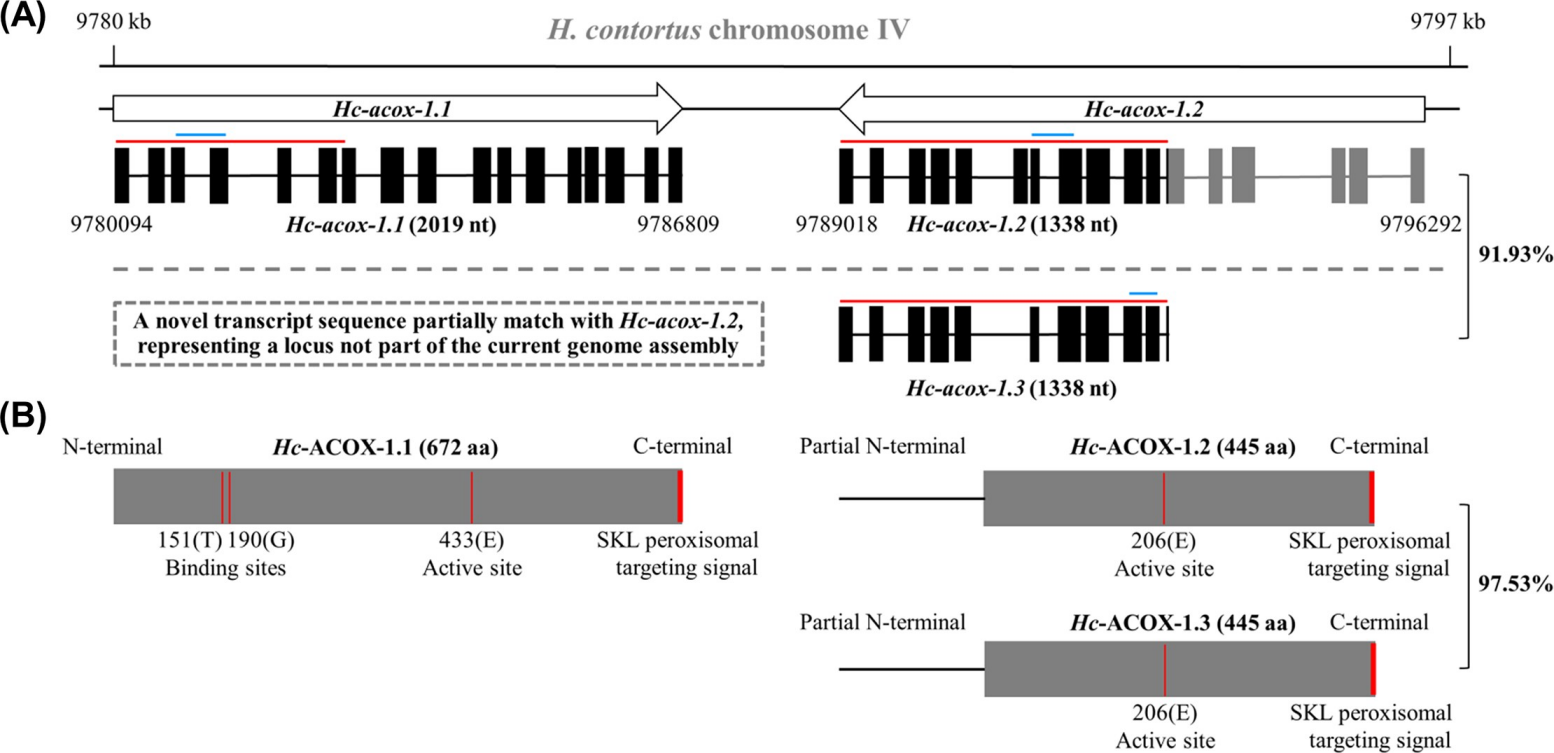
Complementary DNA-confirmed gene models of *acox-1* in *Haemonchus contortus*. (A) Gene structures of *Hc*-*acox-1*.*1* and *Hc-acox-1*.*2* are defined in *H*. *contortus* based on complementary DNA sequences. Arrow boxes indicate two adjacent gene loci of *Hc-acox-1*, with a 91.93% identity indicated between *Hc-acox-1*.*2* and *Hc-acox-1*.*3*, a possible locus not part of the current genome, based on NCBI blastn searching. Black blocks represent exons that were matched with cloned sequences, and horizontal lines represent introns indicated by transcripts. The numbers above and below the boxes indicate the start and end of gene loci. The lines above exons represent the sequences targeted in RNAi (red) and in qPCR (blue), respectively. (B) Predicted key sites in the deduced amino acid sequences of *H*c-ACOX-1.1, -1.2 and -1.3, with a 97.53% similarity indicated between the latter two proteins based on NCBI blastp searching. Predicted flavin adenine dinucleotide binding sites 151 (T, threonine) and 190 (G, glycine), and active site 206/433 (E, glutamic acid) of deduced *Hc*-ACOX-1 are indicated. The amino acid sequences without SKL are used for polyclonal antibodies preparation. SKL represents peroxisomal targeting signal type 1 (PTS1). S: serine, K: lysine, L: leucine.

Three transcripts were identified by mapping molecularly cloned sequences to the reference genome of *H*. *contortus*, matching two gene loci *Hc*-*acox-1*.*1* (chromosome IV: 9780094–9786809) and *Hc*-*acox-1*.*2* (chromosome IV: 9789018–9796292) in chromosome IV of this parasitic nematode (Fig 1A). Specifically, *Hc-acox-1*.*1* was a full-length transcript (2019 nt in length; GenBank accession number: MZ229680), whereas *Hc-acox-1*.*2* (1338 nt in length; GenBank accession number: MZ229681) (Fig 1A). A similar transcript (*Hc-acox-1*.*3*) (1338 nt in length with 91.93% identity to *Hc-acox-1*.*2*; GenBank accession number: MZ229682) was partially matched with the *Hc-acox-1*.*2* gene model, representing a possible locus not part of the current genome assembly (Fig 1A). Three amino acid sequences *Hc*-ACOX-1.1, *Hc*-ACOX-1.2 and *Hc*-ACOX-1.3 were deduced from the gene models of *Hc-acox-1* in *H*. *contortus*. Specifically, two flavin adenine dinucleotide (FAD)-binding sites Thr (151) and Gly (190) and an active site Glu (433) were predicted in *Hc*-ACOX-1.1, with only active site Glu (206) indicated in the deduced partial *Hc*-ACOX-1.2 and *Hc*-ACOX-1.3 (Fig 1B). A peroxisomal targeting signal (PTS1) was predicted at the carboxyl terminal of each deduced *Hc*-ACOX-1 protein (Fig 1B).

### *Hc*-ACOX-1 has palmitoyl-CoA oxidase activity

**Fig 2:**
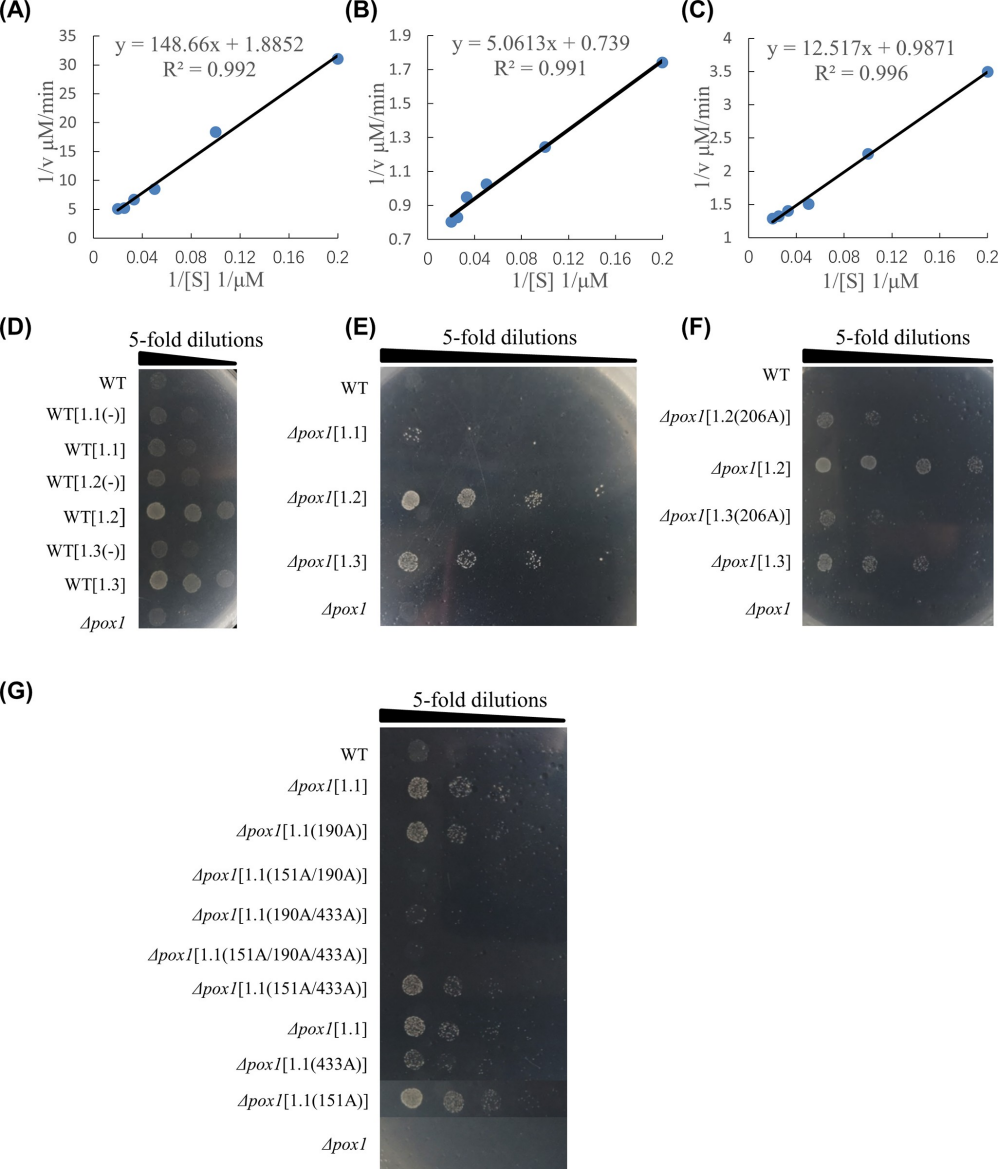
Enzyme activity and spotting assay of *Hc*-ACOX-1 *in vivo* and *in vitro*. (A-C) Linear double-reciprocal plots of *Hc*-ACOX-1.1 (A), *Hc*-ACOX-1.2 (B) and *Hc*-ACOX-1.3 (C) constructed based on reciprocal reaction velocity (1/V) and reciprocal value of palmitoyl-CoA concentration (1/[S]). Panels D-G: The letters (1.1, 1.2 and 1.3) in square brackets represent the corresponding protein of *Hc*-ACOX-1 and the characters in parentheses indicate the key sites that are replaced with Alanine. 151A/190A represents the 151st and 190th amino acids are replaced with Alanine. Yeast concentrations are marked at top with a starting concentration of OD_600_ = 1. (D) Growth of wild-type *Saccharomyces cerevisiae* on YNBO plates (YNB supplemented with oleic acid as sole carbon source). (-) represents mutation without peroxisomal targeting signal type 1. (E) Growth of *S*. *cerevisiae Δpox1* strain rescued with *Hc-acox-1* on YNBO plates. (F) Effect of key site of *Hc*-ACOX-1.2 and -1.3 on the growth of *Δpox1*. (G) Effect of key sites of *Hc*-ACOX-1.1 on the growth of *Δpox1*. Multiple mutations in *Hc*-ACOX-1.1 affect the growth of *Δpox1* on the YNBO plate, whereas single mutation at 151A or 190A did not. WT and *Δpox1* represent wild-type and POX (ACOX homologue) mutant strain of *S*. *cerevisiae*, respectively.

The potential role of *Hc*-ACOX-1 in fatty acid β-oxidation was evaluated by testing the palmitoyl-CoA oxidase activity of the recombinant protein *in vitro* (S1A Fig). Specifically, optimum temperatures for the activity of r*Hc-*ACOX-1.1, -1.2 and -1.3 were determined by measuring activity at 37°C, 42°C and 42°C, respectively, and a consistent optimum pH at 7.5 (S1C and S1D Fig). Under optimum conditions, r*Hc-*ACOX-1.1, -1.2 and -1.3 showed different potencies of oxidase activity, with maximum reaction rate (Vmax) measured at 0.53 μM/min, 1.35 μM/min and 1.01 μM/min, respectively, and Michaelis constant (Km) at 78.79 μM, 6.83 μM and 12.64 μM, respectively (Fig 2A and 2C). The function of *Hc*-ACOX-1 in fatty acid oxidation was further confirmed in *Saccharomyces cerevisiae*. Specifically, supplementation of oleic acid in the culture medium promoted the growth of wild type yeast expressing *Hc*-ACOX-1, and the deficiency of *Δpox1* (peroxisomal acyl-CoA oxidase loss-of-function strain) in utilising oleic acid and growing in YNBO medium (YNB medium supplemented oleic acid as the sole carbon source) was rescued by expressing *Hc*-ACOX-1, with different potencies detected for the three proteins (Fig 2D and 2E). Deleting the PTS1 of *Hc*-ACOX-1 led to compromised growth of wild-type yeast in YNBO medium (Fig 2D). Introducing site (i.e., binding and active sites) mutations into the amino acid sequences of *Hc*-ACOX-1 compromised the growth of *Δpox1* on the YNBO plate. However, mutations at Thr (151) and Glu (433) in *Hc*-ACOX-1.1 showed weak effects on the growth of *Δpox1* (Fig 2F and 2G). Although single mutation in *Hc*-ACOX-1.1 (151A or 190A) did not show obvious effect on the growth of *Δpox1* on the YNBO plate, multiple mutations compromised the growth of *Δpox1* (Fig 2G).

### *Hc*-ACOX-1 interacts with *Hc*-PEX-5 in peroxisomes

**Fig 3:**
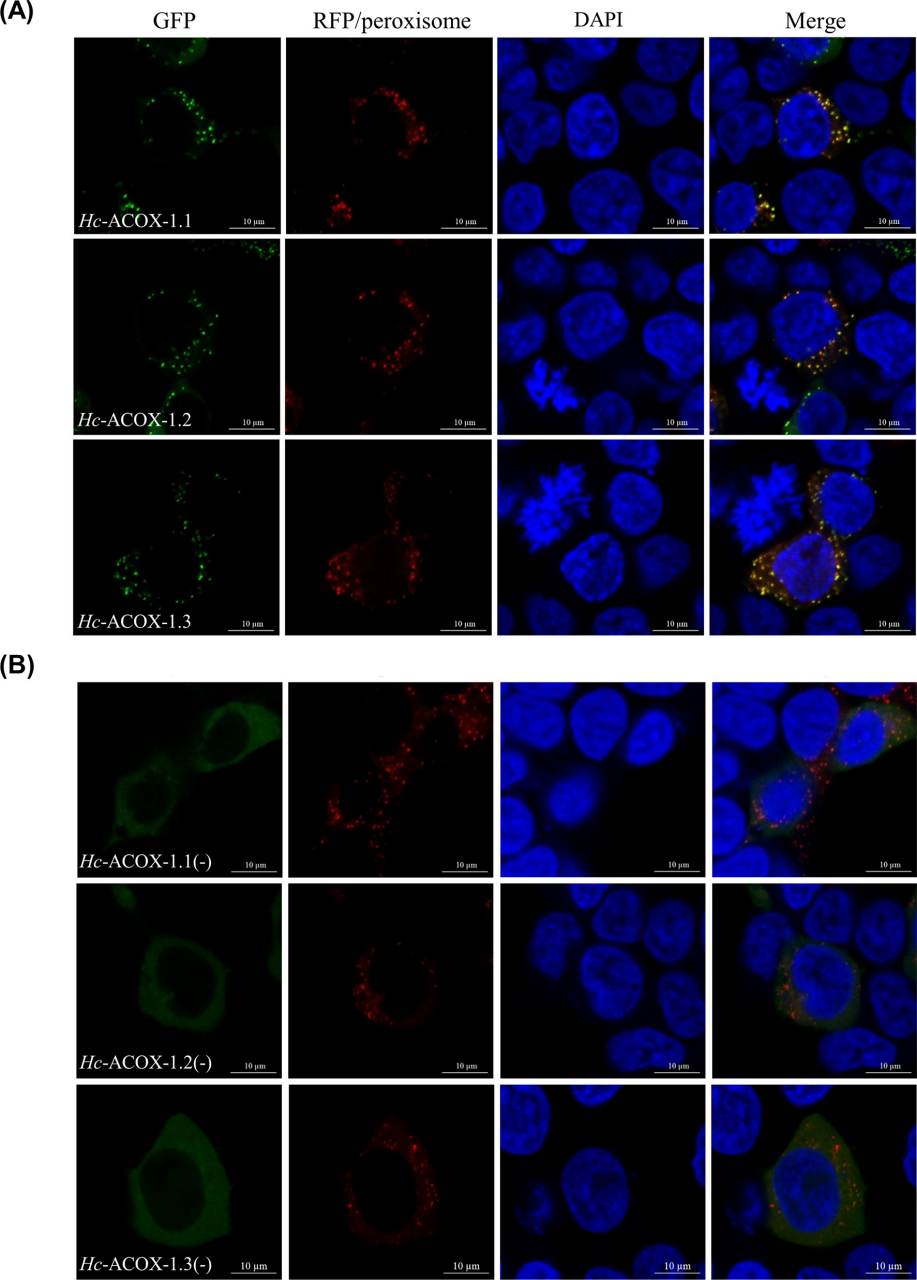
Co-localisation of *Hc*-ACOX-1 and peroxisomes in HEK293T cells. (A) Subcellular localisation of *Hc*-ACOX-1 and peroxisomes in HEK293T cells. (B) Subcellular localisation of *Hc*-ACOX-1 without peroxisomal targeting signal type 1 (PTS1) and peroxisomes in HEK293T cells. *Hc*-ACOX-1 without PTS1 is designated as *Hc*-ACOX-1 (-). Green fluorescent protein (GFP)-fused ACOX-1 is expressed in HEK293T cells and the nuclei are stained with 4’,6-diamidino-2-phenylindole (DAPI). Red fluorescence indicates RFP/peroxisome protein expressed in peroxisome. Scale bar: 10 μm.

**Fig 4:**
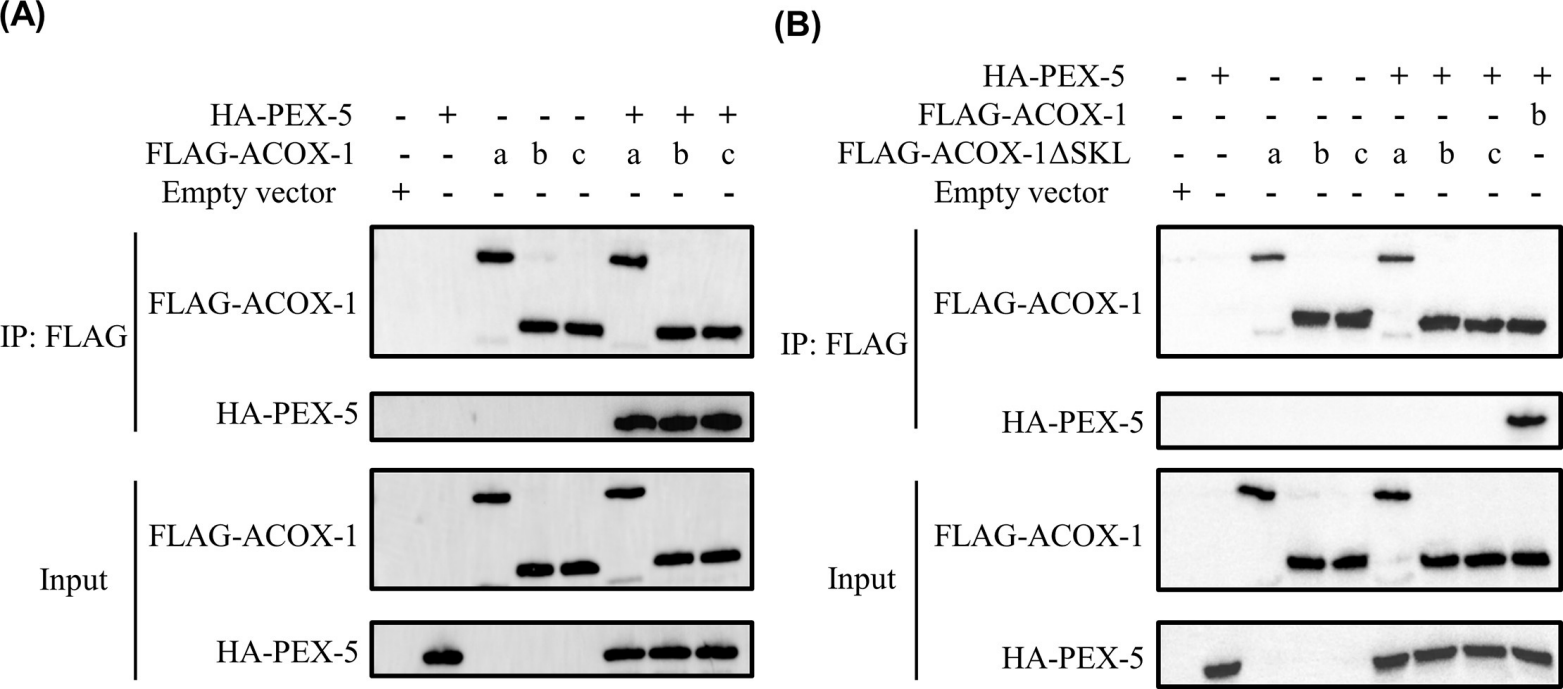
Interaction of *Hc*-ACOX-1 and *Hc*-PEX-5 via peroxisomal targeting signal type 1 (PTS1). (A-B) Co-expression of *Hc-*ACOX-1-FLAG with (A) or without (B) PTS1 and HA-tagged *Hc*-PEX-5 in HEK293T cells. Anti-FLAG antibody is used in immunoprecipitation (IP, top two panels) of the total cellular lysates (input, bottom tow panels). The immunoprecipitated portion and input are individually subjected to Western blot using anti-DYKDDDDK (FLAG) tag and anti-HA tag antibodies as labelled. The letters a, b and c represent *Hc*-ACOX-1.1, -1.2, and -1.3, respectively. *Hc*-ACOX-1.2 is used as positive control in (B). ΔSKL represents the deletion of PTS1.

A punctate distribution was observed for *Hc*-ACOX-1 protein in the cytoplasm of HEK293T cells (Fig 3A), which colocalised with peroxisomes, rather than mitochondria (S2 Fig). Without a PTS1 sequence, *Hc*-ACOX-1 could not be translocated to peroxisomes and was homogenously distributed in the cytoplasm (Fig 3B). The peroxisomal distribution of *Hc*-ACOX-1 indicated potential interactions between *Hc*-ACOX-1 and peroxins, which was verified by the growth of positive yeast clones expressing *Hc*-ACOX-1 and *Hc*-PEX-5 on QDO plates (quadruple dropout supplements, SD/-Leu/-Trp/-His/-Ade) in the yeast two hybrid (Y2H) assay (S3 Fig). By contrast, yeast expressing *Hc*-ACOX-1 without PTS1 could not grow on QDO plates, in accordance with the untargeted peroxisomal distribution of PTS1-lossing *Hc*-ACOX-1 (S3 Fig). In addition, an interaction between flag-tagged *Hc*-ACOX-1 and HA-tagged *Hc*-PEX-5 was confirmed in a co-immunoprecipitation (Co-IP) assay, and PTS1 also played an essential role in the recognition of *Hc*-ACOX-1 by *Hc*-PEX-5 (Fig 4).

### *Hc*-ACOX-1 is expressed in the intestine and hypodermis

**Fig 5:**
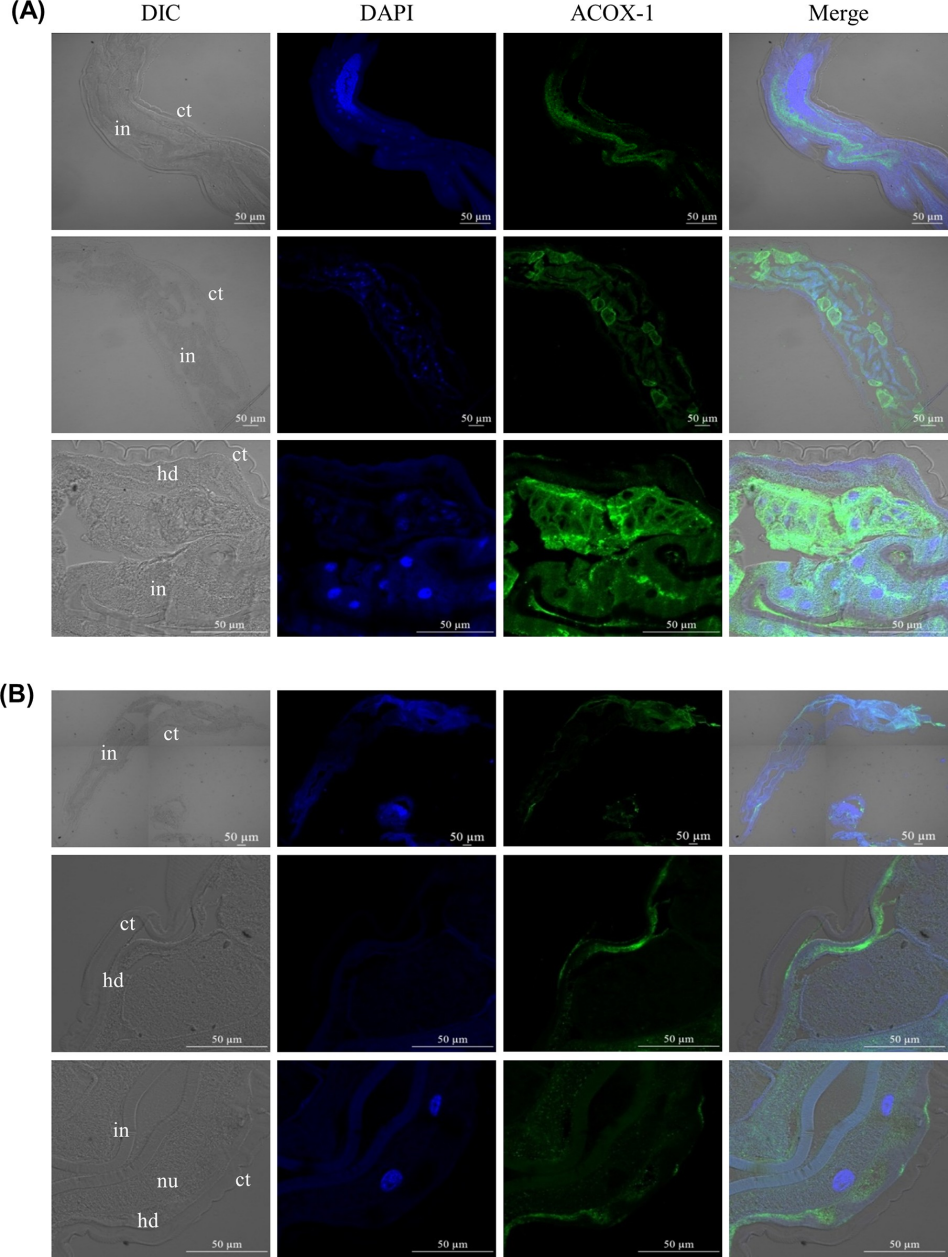
Tissue immunolocalisation of *Hc*-ACOX-1 in the fourth-stage larvae (L4s) and adult of *Haemonchus contortus*. Tissues of L4s (A) and female adults (B) of *H*. *contortus* are probed with rabbit anti-r*Hc*-ACOX-1.1 polyclonal antibodies followed by fluorescein conjugated-goat anti-rabbit IgG (H+L) as secondary antibody. Nuclei are counterstained with 4’,6-diamidino-2-phenylindole (DAPI). DIC: differential interference contrast, ct: cuticle, in: intestine, hd: hypodermis, nu: nucleus. Sections incubated with pre-immune serum (negative control) are provided in supplementary S5B Fig. Scale bar: 50 μm.

Polyclonal antibodies generated against each recombinant protein r*Hc-*ACOX-1.1, -1.2 and -1.3 recognised the native *Hc*-ACOX-1 proteins of *H*. *contortus*, but could not distinguish the proteins (S4 Fig). Using the polyclonal antiserum (anti-r*Hc-*ACOX-1.1), protein distribution of *Hc*-ACOX-1 was shown to predominate in the intestine and hypodermis of L4s (Fig 5A) and the adults of *H*. *contortus* (Figs 5B and S5A).

### Developmental transcription of *Hc*-ACOX-1 coding gene

**Fig 6:**
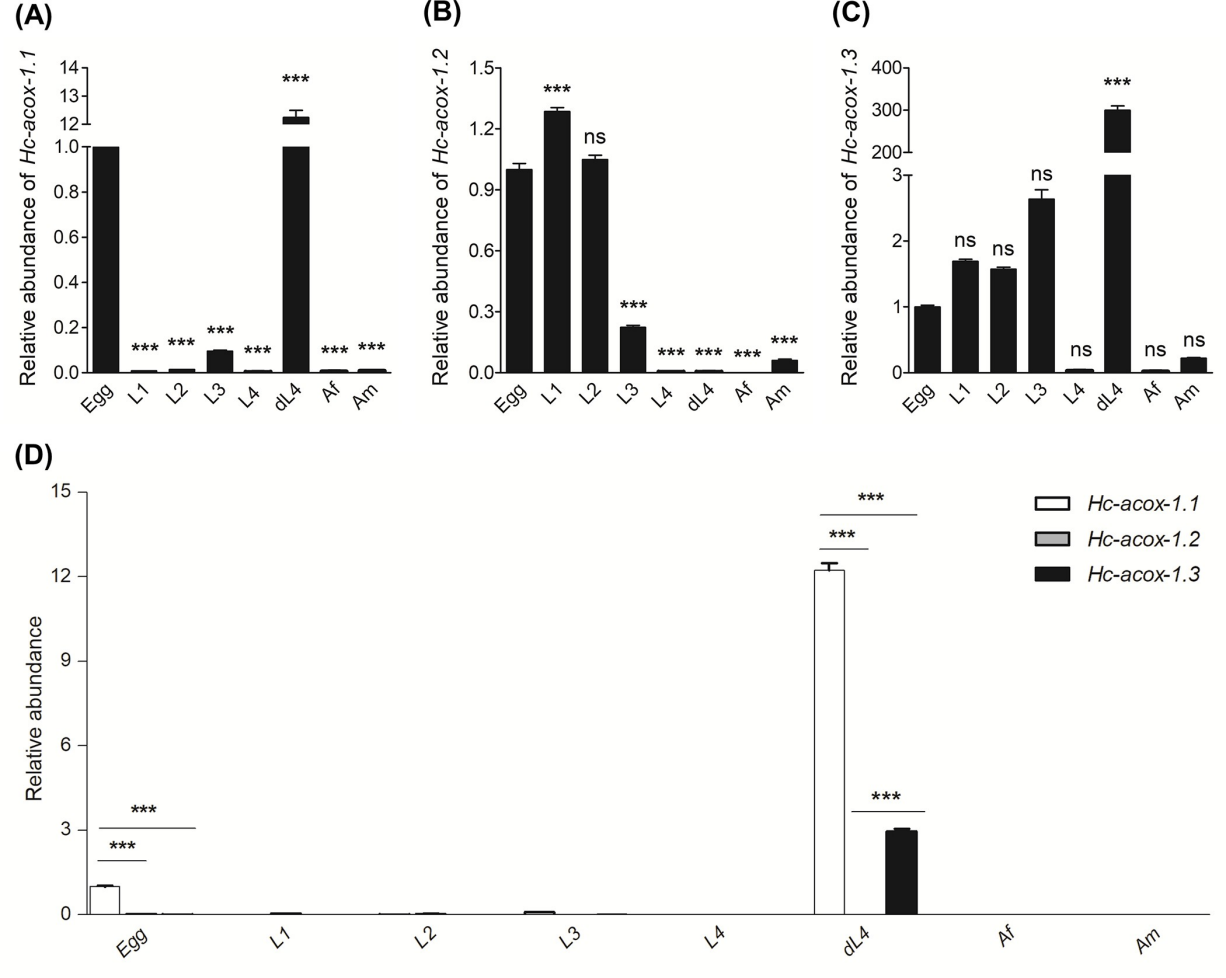
Transcriptional dynamics of *Hc-acox-1* in different life cycle stages of *Haemonchus contortus*. Transcripts of *Hc-acox-1*.*1* (A), *Hc-acox-1*.*2* (B) and *Hc-acox-1*.*3* (C) in the egg, first- (L1), second- (L2), third- (L3), fourth-larval (L4) stages, diapaused L4 (dL4) and adult female (Af) and male (Am) of *H*. *contortus* were detected by quantitative real-time PCR using *Hc*-*β*-*tubulin* as an internal control. Expressional level of *Hc-acox-1* at the egg stage is set as one unit, and those of the other life cycle stage are relative to eggs. (D) Relative expression levels of three transcripts compared with each other in different stages of *H*. *contortus*. The statistical analysis was performed using 2^−ΔΔCt^ method in Excel 2016 and one-way ANOVA with Dunnett post-hoc test in GraphPad Prism 5. All Data are resented by mean ± SEM. Three technical replicates are included for three independent experiments. Statistical analysis is performed using one-way ANOVA with Dunnett post-hoc test. *P<0.05, **P<0.01, ***P<0.001, ns: no significance.

Different transcriptional patterns were observed for the three transcripts *Hc-acox-1*.*1*, *-1*.*2* and *-1*.*3* among key developmental stages of *H*. *contortus*. Specifically, *Hc-acox-1*.*1* was highly transcribed in the egg and diapause stages, particularly in the latter (Fig 6A); *Hc-acox-1*.*2* was predominantly transcribed in the egg, L1, L2 and L3 stages collected from the environment while transcribed at low level in the diapause stage collected from the host animals (Fig 6B); *Hc-acox-1*.*3* was highly transcribed in the early developmental stages (i.e., egg, L1, L2 and L3), with a relatively higher transcriptional level detected in the diapause stage of *H*. *contortus* (Fig 6C). None of the three transcripts was highly expressed in the L4 and adult female and male of this parasitic nematode (Fig 6A–6C). The relative transcriptional level of *Hc-acox-1*.*1* in egg and diapaused L4 stage was significantly higher than that in other stages (Fig 6D).

### Knockdown of *Hc-acox-1* increases larval death

**Fig 7:**
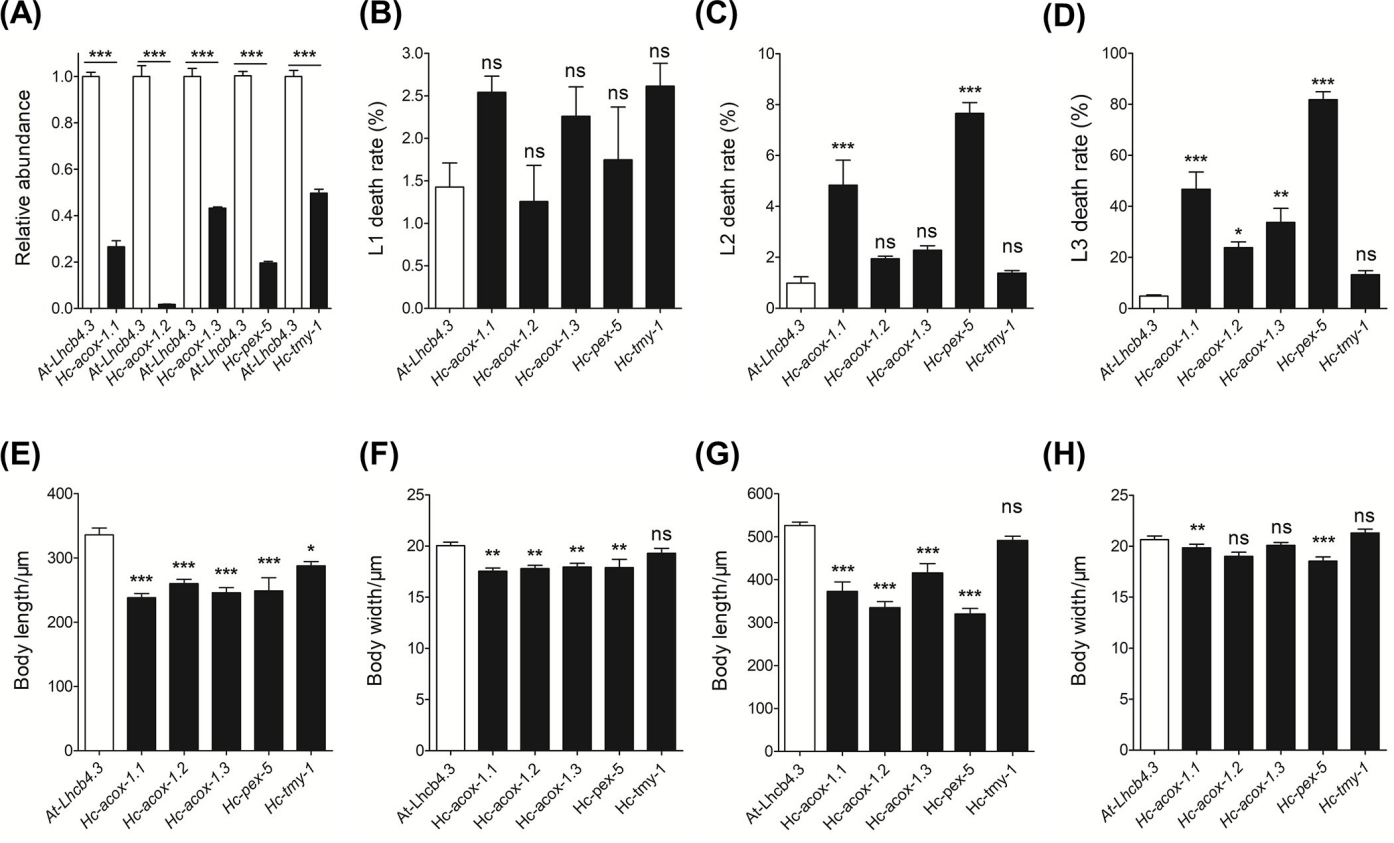
Effects of gene knockdown of *acox-1* and *pex-5* on *Haemonchus contortus* larval development and survival. (A) Relative transcriptional levels of *Hc-acox-1*.*1*, *-acox-1*.*2*, *-acox-1*.*3* and -*pex-5* in RNA interference (RNAi)-treated worms. *Hc*-*β*-*tubulin* is used as an internal control. *Arabidopsis thaliana* light harvesting complex gene (*Lhcb4*.*3*) is used as a negative control. *H*. *contortus* tropomyosin (*Hc-tmy-1*) is used as a positive control. The relative abundance of each transcripts in treated worms is compared with that of the negative control. (B-D) Death rates of the first- (L1, B), second- (L2, C) and third- (L3, D) stage larvae of RNAi-treated worms. (E-F) Changes in body length (E) and body width (F) of RNAi-treated L2s of *H*. *contortus*. (G-H) Changes in body length (G) and body width (H) of RNAi-treated L3s of *H*. *contortus*. Data in all panels are presented in mean ± SEM (n = 20). Statistical analysis is performed using one-way ANOVA with Dunnett post-hoc test. *P<0.05, **P<0.01, ***P<0.001, ns: no significance.

*H*. *contortus* larvae were fed on or soaked in the bacteria expressing dsRNA of *Hc-acox-1* and *Hc-pex-5*. Transcriptional levels of these genes were significantly (*P* < 0.001) reduced in the treated worms than untreated controls (Fig 7A). Compared with negative control, knockdown of *Hc-acox-1* (particularly *Hc-acox-1*.*1*) resulted in an increase in death of L2s and L3s (Fig 7B and 7D), and a decrease in body length (*P* < 0.001) and width (*P* < 0.05) of L2s (Fig 7E and 7F) and L3s (Fig 7G and 7H) of *H*. *contortus*. In particular, knockdown of *Hc-pex-5* led to a lethal phenotype at the late L2 stage of *H*. *contortus* (Fig 7C and 7D), with body length and width significantly decreased (*P* < 0.05) (Fig 7E and 7F).

## Discussion

In this work, we identified a key gene, *Hc-acox-1*, that is likely involved in the peroxisomal fatty acid β-oxidation pathway of *H*. *contortus*, a parasitic nematode of economic significance worldwide. *Hc-acox-1*.*1*, *-1*.*2* and *-1*.*3* encode three proteins, which all show a fatty acid oxidation activity and an interaction with PEX-5 in peroxisomes. *Hc-acox-1* is predominantly detected in the intestine and hypodermis of *H*. *contortus*, particularly in the early larval stages and the diapaused L4s. Gene knockdown analysis indicates that *Hc-acox-1* and *Hc-pex-5* are crucial for early larval development and likely in the facultative developmental arrest (hypobiosis) of *H*. *contortus*.

Identification of the previously unknown *Hc-acox-1* fills a gap in the peroxisomal fatty acid β-oxidation pathway in parasitic nematodes, particularly *H*. *contortus*. Peroxisomal β-oxidation participates in lipid metabolism by oxidizing long-chain fatty acids and branched-chain fatty acids, which is crucial for the biosynthesis of dauer pheromones, short-chain ascarosides secreted by the free-living nematode *C*. *elegans* in response to environmental variants [4,16,44,45]. Although ascarosides have been identified in a broad range of free-living and parasitic nematodes and might play roles in mediating a variety of behaviours [30,46], little is known about these aspects in *H*. *contortus* and related species of the order Strongylida–one of the largest groups of pathogenic worms in animals. In addition, specific ascaroside profiles were also indicated among species, suggesting species specificity of ascaroside synthesis and signalling among nematodes [47]. However, little is known about mechanisms underlying the species specificity of ascaroside biosynthesis in parasitic nematodes. In our previous work [37–40], three (*maoc-1*, *dhs-28* and *daf-22*) of four genes that are known to be involved in the fatty acid β-oxidation have been identified in *H*. *contortus*, which implies a relatively conserved fatty acid β-oxidation pathway between this parasitic nematode and the model organism *C*.*elegans*. In the current work, identification of the last gene homologue *Hc-acox-1* confirmed the genetic basis for fatty acid β-oxidation cycles and short-chain ascaroside biosynthesis in this parasitic nematode. On this genetic basis, experiments for detecting specific ascarosides and exploring their biological roles in *H*. *contortus* and related parasitic nematodes can be performed with greater confidence.

Our heterologous expression studies support that *Hc-*ACOX-1 plays a role in fatty acid oxidation in peroxisomes by interacting with *Hc-*PEX-5. Although encoded by three transcripts of two genes, enzymatic activities of all the three proteins in fatty acid oxidation have been verified both *in vitro* and *vivo*. Interestingly, *Hc*-ACOX-1.2 and *Hc*-ACOX-1.3 proteins without FAD-binding site showed higher enzymatic activities, suggesting a likely regulatory role of this binding-site in the fatty acid oxidation, which should be further investigated. In particular, heterologous expression of *Hc-*ACOX-1 rescued the deficiency of *Δpox1* strain of *S*. *cerevisiae* in utilising oleic acid and promoted the growth of wild-type *S*. *cerevisiae*. Importantly, we found that the peroxisomal targeting signal (PTS1) was essential for the acyl-CoA oxidase activity of *Hc-*ACOX-1. Without the PTS1 sequence, *Hc-*ACOX-1 was homogenously distributed in the cytoplasm and could not be translocated into the peroxisomes [48,49], which explains the failed rescue of *Δpox1* strain of *S*. *cerevisiae* using a PTS1-lossing mutant [50,51]. The essential role of PTS1 was further confirmed by Y2H and Co-IP assays, in which *Hc-*ACOX-1 is recognised by *Hc-*PEX-5 via the PTS1 [51]. Peroxins (PEX) are required for peroxisome biogenesis [52], particularly in importing peroxisomal matrix proteins synthesised in the cytosol into the peroxisomes [53]. Proteins with a PTS1 can be recognised by PEX which serves as a receptor for matrix-targeted proteins [54–57]. From this, we conclude that function of *Hc-*ACOX-1 in peroxisome is *Hc-*PEX-5-dependant, suggesting a class of potential intervention targets of fatty acid oxidation in *H*. *contortus*.

ACOX-1 and PEX-5 might play important roles in the post-embryonic larval development and facultative developmental arrest of parasitic nematodes. This statement can be supported firstly by the developmental transcription analysis of *Hc-acox-1*, which showed high mRNA levels of specific spliced variants in the early larval stages (free-living stages in the environment) of *H*. *contortus*, as well as in the diapause stage (early parasitic stage in the host animals) of this nematode. It is likely a molecular response in the free-living larvae to the environmental variants, as there is no stable food availability, temperature and moisture before entering into a host. It is also a possible molecular alteration for facultative developmental arrest of L4s within host animals, particularly when seasonal and host immune or physiological conditions are unfavourable for a continuous development of this parasitic nematode [58]. In addition, the potential roles of ACOX-1 and PEX-5 in early larval development and developmental arrest of *H*. *contortus* are also supported by the gene knockdown analyses. For instance, knockdown of *Hc-acox-1* and *Hc-pex-5* resulted in significantly changed body length and width of the L2s and L3s of *H*. *contortus*.

There are also some questions pertaining to *acox-1* and *pex-5* that still need to be further investigated in *H*. *contortus* and related parasitic nematodes. In particular, the different transcriptional patterns of spliced variants of *Hc-acox-1* and their specific functions (e.g., synthesis of ascarosides with specific side-chain lengths) are not clear. Knockdown of *Hc-pex-5* resulted in a lethal phenotype in *H*. *contortus* at the second larval stage, which is of major interest to be understood in detail as it might play multiple roles in the essential biological processes of this parasitic nematode. Additionally, little is known about the functions (particularly the essentiality) of *Hc-acox-1* and *Hc-pex-5* gene homologues in related species of the order Strongylida, and other socioeconomically important parasitic nematodes. Moreover, functional interactions between ACOX-1 and other peroxins, as well as functional relationships among different ACOXs warrant further investigation [35,59]. A better understanding of these aspects should provide insights into the developmental biology of parasitic worms and the discovery of novel targets to control major parasitic diseases.

In conclusion, we identified a palmitoyl-CoA oxidase ACOX-1 in *H*. *contortus*, which appeared to be required for normal post-embryotic larval development in the environment and likely in hypobiosis within host animals. Perturbation of this molecule and its interacting protein PEX-5 results in shortened lifespan and even lethality in *H*. *contortus*, suggesting novel potential targets for the control of parasitic diseases of socioeconomical significance.

## Materials and methods

### Ethics statement

The use of sheep and rabbits in this work was approved by the Experimental Animal Ethics Committee, Zhejiang University (Number: 20170177). All animals were cared for according to the Regulation for the Administration of Affairs concerning Experimental Animals of the People’s Republic of China.

### Nematode maintenance

*H*. *contortus* (ZJ strain) was maintained in Hu sheep as described elsewhere [60,61]. Briefly, 6-month-old helminth-free male sheep were experimentally infected with ~ 8000 infectious larvae per os. Eggs were isolated from feces by flotation using saturated NaCl and incubated on (Luria-Bertani) LB agar plates at 28°C. The first- (L1s), second- (L2s), and third-stage larvae (L3s) were collected after incubation for 1, 3 and 7 days, respectively. The fourth-stage larvae (L4s) were obtained from the abomasum of sheep 9 days post infection. Adult (female and male) worms were collected from the abomasum 45 days after infection. Diapaused worms were obtained from the abomasum after the lambs were held for 60 days after infection in winter.

### Molecular cloning and sequence analysis

Total RNA was extracted from *H*. *contortus* adult worms using Trizol reagent (Invitrogen, Carlsbad, CA, USA), and the first-strand cDNA was synthesized employing a cDNA synthesis kit (Toyobo, Japan). *Hc-acox-1* was amplified using three primer sets (S1 Table) that were designed using Primer Premier 5 software (Premier Biosoft International, Palo Alto, CA, USA) based on genomic and transcriptomic information of *H*. *contortus* [62,63]. Reaction mixes for PCR amplification contained 2.5 μL 10 × PCR buffer, 2 μL dNTPs (2.5 mM), 1 μL forward primer (10 μM), 1 μL reverse primer (10 μM), 1 μL cDNA, 17 μL molecular grade water and 0.5 μL LA Taq (Takara, Japan). The thermocycling program comprised an initial denaturation at 95°C for 5 min, 30 cycles of denaturation at 95°C for 15 s, annealing at 55°C for 30 s, extension at 72°C for 2 min, and a final extension at 72°C for 10 min. PCR products were ligated to the pMD18-T vector (Takara, Japan) and sequenced.

The obtained nucleotide sequences were manually inspected by searching against WormBase ParaSite (https://parasite.wormbase.org) and UniProtKB (https://www.uniprot.org/uniprot) databases. Transcript variants were confirmed based on homology searching, multiple sequence alignment analysis and genome mapping. Confirmed sequences were used for gene model validation and potential curation, based on the reference genome (PRJNA205202.WBPS15) of *H*. *contortus* (haemonchus_contortus_MHCO3ISE_4.0 in WormBase ParaSite). The validated gene model of *acox-1* in *H*. *contortus* was compared with that of *C*. *elegans*.

### Enzyme activity assay

A Kozak sequence and a FLAG tag sequence were added to the N-terminus of each *Hc-acox-1* splice variant using a two-round PCR approach. Each recombinant protein was cloned into the pcDNA3.1 vector, which was then used to transfect HEK293T cells. Transfected cells were harvested for Western blot analysis and protein purification. In brief, the harvested cells were lysed in a lysis buffer (10 mM NaF, 25 mM glycerol 2-phosphate, 1 mM Na_3_UO_4_, 200 mM NaCl, 25 mM Tris, 1% Nonident P-40) supplemented with a protease inhibitor cocktail (Bimake, Houston, TX, USA) with constant shaking at 4°C for 15 min. The lysate was centrifuged at 12000 × *g* for 10 min and the recombinant *Hc*-ACOX-1 (r*Hc*-ACOX-1) was isolated from the supernatant using anti-flag magnetic beads (Bimake, USA). Protein purity was examined by SDS-PAGE.

Activity of the r*Hc*-ACOX-1 expressed in HEK293T cells was determined by measuring substrate [Palmitoyl-Coenzyme A]-dependent H_2_O_2_ production in a horseradish peroxidase (HRP) catalysed oxidative coupling reaction. Briefly, 0.05 μg/μL *Hc*-ACOX-1 protein, 50 mM potassium phosphate, 0.82 mM 4-aminoantipyrine, 10.6 mM phenol, 10 μM FAD, 5 IU horseradish peroxidase and 20 μM palmitoyl-CoA were used in a 200 μL reaction mixes. H_2_O_2_ production in the reaction was measured for 5 min by recording the absorbance at 490 nm every 10 s using a Chro Mate spectrophotometer (Awareness Technology, USA), at different temperatures and pH. Maximum reaction rate (Vmax) and Michaelis constant (Km) were calculated.

### Spotting assay

Spotting assay was employed for the analysis of active sites of *Hc*-ACOX-1 as described previously [34]. Site mutations were introduced into the *Hc-acox-1* sequence by PCR amplification to replace Thr (151), Gly (190) and Glu (433) in *Hc*-ACOX-1.1 with Ala, and Glu (206) in *Hc*-ACOX-1.2 and *Hc*-ACOX-1.3 with Ala. A 6× His tag sequence was also added to the N-terminus of each *Hc-acox-1* mutant. Each *Hc-acox-1* mutant was inserted into the VTU260 plasmids via *Nhe* I and *BamH* I sites, and transformed into the *Δpox1* mutant strain of *S*. *cerevisiae* (YGL205W). The transformed *Δpox1* strain of *S*. *cerevisiae* was synchronized in a YNB medium (0.67% yeast nitrogen base, Invitrogen, Carlsbad, CA, USA) and diluted to a concentration of OD_600_ = 1. Synchronized *S*. *cerevisiae* were spotted on YNBO agar plates containing 0.2% oleic acid (Sigma-Aldrich, St. Louis, MO, USA). The spotting assay was performed after 48 hours of incubation at 30°C. A wild-type strain of *S*. *cerevisiae* was used as a control.

### Co-transfection and co-localisation

Each of the three variants of *Hc-acox-1* and their mutants was cloned into pEGFP-C2 vectors via *Hind* III and *BamH* I restriction enzyme sites using a one-step PCR cloning kit (Novoprotein, Shanghai, China) following the supplier’s instructions. HEK293T cells were transiently transfected with the recombinant pEGFP-C2-*Hc-acox-1* plasmids with Lipofectamine 2000 (Invitrogen, USA). HEK293T cells were also transfected with mCherry-Mito-7 (Addgene plasmid # 55102) or mCherry-Peroxisomes-2 (Addgene plasmid #54520) plasmids to ascertain localisation of *Hc*-ACOX-1 to mitochondria or peroxisomes. After 24 hours of incubation, cells were harvested for Western blot analysis and fluorescence observation using a fluorescence microscope (Zeiss, Germany). In brief, cells grown on coverslips were fixed in ice-cold 4% paraformaldehyde in phosphate buffered saline (PBS) (pH 7.4) at 4°C for 15 min and then washed in PBS, followed by permeabilization with 0.1% Triton X-100 in PBS for 5 min. After three washes, cells were incubated with 0.1 μg/mL 4’,6-diamidino-2-phenylindole (DAPI) at 37°C for 15 min and then observed with a Zeiss LSM710 laser confocal microscope (Zeiss, Germany).

### Yeast two-hybrid (Y2H) and co-immunoprecipitation (Co-IP)

A Matchmaker Gold Yeast Two-Hybrid (Y2H) System (Takara, Japan) was used to investigate the potential interaction of *Hc*-ACOX-1 and peroxins (PEXs) *in vitro*. In brief, *Hc-acox-1* and PEX-coding sequences (*Hc*-*pex-1*, *3*, *5* or *19*) were cloned into pGBKT7 and pGADT7 vectors, and transformed into Y2HGold and Y187 yeast strains, respectively. Colonies of Y2HGold yeast expressing bait protein ACOX-1 and Y187 expressing prey protein PEX were incubated in 5 mL of 2×YPDA liquid medium supplemented with 50 μg/mL kanamycin with shaking (30 rpm) at 30°C for 24 h. The culture was centrifuged at 1000 × *g* for 10 min and cells were then suspended in 1 mL physiological saline (0.9% NaCl). All cells were inoculated on SD/-Trp or SD/-Leu plates at 30°C for 3–5 days. After mating, the colonies grown on DDO (SD/-Trp/-Leu) and QDO (SD/-Leu/-Trp/-His/-Ade) plates were transferred onto QDO (SD/-Leu/-Trp/-His/-Ade) agar plates supplemented with X-α-gal and Aureobasidin A.

Co-IP was also performed to examine the potential interactions of *Hc*-ACOX-1 and *Hc*-PEXs. Briefly, both *Hc-acox-1* (fused with FLAG-tagged sequence) and *Hc*-*pex-1* (or *-3*, *-5*, *-19*) (fused with HA-tagged sequence) were cloned into a pcDNA3.1 vector and transfected into the HEK293T cells. Cultured cells were lysed, and the lysate was incubated with 20 μL anti-flag beads at 4°C overnight. Purified proteins were subjected to 12% SDS-PAGE and Western blot analysis. Anti-FLAG tag and anti-HA tag rabbit serum (1:1000, Cell Signaling Technology, Danvers, MA, USA) were used as the primary antibodies, and horseradish peroxidase-conjugated goat anti-rabbit IgG was used as the secondary antibody (1:5000). The chemical signal was detected using a FDbio-Dura ECL kit (FDbio, China).

### Preparation of polyclonal antibodies

Each of the three variants of *Hc-acox-1* was inserted into the pCold II vector via *BamH* I and *Hind* III restriction enzyme sites. Recombinant plasmids were individually transformed into BL21 (DE3) cells, and the expression of recombinant protein (r*Hc-*ACOX-1) was induced by 0.2 mM isopropyl β-D-1-thiogalactopyranoside (IPTG) at 16°C overnight. Cultured cells were harvested and suspended in 50 mM PBS supplemented with proteinase inhibitors (Fdbio, China), then sonicated for 45 min in an ice bath using a 2D ultrasonic cell disruptor (Scientz, Ningbo, China). The supernatant was separated by centrifugation at 12000 × *g* at 4°C for 10 min and then purified with a Ni-NTA resin column (Thermo Fisher Scientific, Waltham, MA, USA). Recombinant protein was eluted from the column with 250 mM imidazole, and checked by SDS-PAGE. Rabbit anti-r*Hc*ACOX-1 polyclonal antibodies were prepared using a method described previously [64,65]. The titre of polyclonal antibodies was determined by an enzyme-linked immunosorbent assay (ELISA) as described previously [66]. Naïve serum collected prior to immunization was used as a negative control.

### Fluorescent immunohistochemistry

L4s of *H*. *contortus* were washed in PBS, then fixed in 4% paraformaldehyde for 24 h, dehydrated, embedded in paraffin, and sectioned at 5 μm in thickness. Worm sections were dried in an oven at 60°C overnight, deparaffinized with xylene and dehydrated in a serial gradient of ethanol. Sections were examined for tissue integrity under a microscope (Zeiss, Germany) by hematoxylin and eosin (H&E) staining. Prepared slides were blocked with 50 μL 1% BSA/PBS for 2 h, washed in PBST, and incubated with antiserum (anti-r*Hc-*ACOX-1.1) using a 1:500 dilution at 4°C overnight. After five washes, slides were then incubated with goat anti-rabbit IgG (H+L) highly cross-adsorbed secondary antibodies (Fluor Plus 488, Thermo Fisher, USA) at a 1:1000 dilution for 1 h. Slides were further washed and incubated with 0.1 μg/mL DAPI at 37°C for 15 min, then examined under a Zeiss LSM710 laser confocal microscope (Zeiss, Germany). The pre-immunisation serum of rabbits was used as negative controls.

### Quantitative real-time PCR

Total RNA extraction and cDNA synthesis were conducted for the egg, L1, L2, L3, L4 and adult stages as well as the diapaused stage of *H*. *contortus*. Transcriptional levels of *Hc-acox-1* in all the developmental stages of *H*. *contortus* were measured by quantitative real-time PCR using a CFX96 real-time PCR system (Bio-Rad, Hercules, CA, USA). Total volume of each reaction was 20 μL (10 μL SYBR green master mix, 0.8 μL forward primer, 0.8 μL reverse primer, 7.4 μL molecular grade water and 1 μL cDNA) and the thermocycling program consisted of 95°C for 60 s, 40 cycles of 95°C for 15 s, 60°C for 15 s and 72°C for 45 s. The melt curve stage was 95°C for 10 s, 60°C for 5 s and 95°C for 5 s. *Hc*-*β*-*tubulin* was selected as an internal control. Primer sets used in this section were shown in S1 Table 2^−ΔΔCt^ method was used for the transcriptional analysis of genes. Three biological and technical replicates were included for statistical analysis.

### RNA interference (RNAi)

Gene knockdown of *Hc-acox-1* and *Hc-pex-5* were conducted in *H*. *contortus* using a soaking method as described before [67]. In brief, HT115 (DE3) bacteria were transformed with the L4440-*Hc-acox-1* or L4440-*Hc-pex-5* vector (expressing double stranded RNAs), cultured and resuspended in physiological saline with an OD of 0.23–0.24. Antibiotic-antimycotic-treated eggs (~ 3000 eggs) were incubated with the transformed HT115 (DE3) cells in culture medium (Earle’s balanced salt solution and 1% yeast extract) supplemented with carbenicillin (100 μg/mL), amphotericin B (2 μg/mL) and 5-fluorocytosine (5 μg/mL), in 6-well culture plates at 28°C for 7 days. *Hc-tropomyosin* and *Arabidopsis thaliana* light harvesting complex gene (*Lhcb4*.*3*) were used as positive and negative control, respectively. Untreated larvae were used as a blank control. All worms were collected for gene knockdown analysis using qRT-PCR, with *Hc*-*β*-*tubulin* selected as an internal control. Primer sets used in this section were shown in S1 Table.

### Statistical analysis

At least three technical replicates were included in each assay and each experiment was repeated three times. Data are presented as mean ± standard error of mean (SEM). One-way ANOVA with Dunnett post-hoc test was performed using Excel 2016 (Microsoft, WA, USA) and GraphPad Prism 5 (San Diego, CA, USA). *P < 0*.*05* was considered statistically significant.

## Supporting information

S1 Table List of primers used in this study. (DOCX)

S1 Fig Construction of enzymatic activity system *in vitro*. (A) Validation of eukaryotic expression of *Hcdacox-1* in HEK293T cells for the purity using SDS-PAGE. (B) Construction of standard curve using hydrogen peroxide as substrate. (C-D) Determination of optimum temperature (C) at pH 7.4 and optimum pH value (D) at 30°C using palmitoyl-CoA as substrate. Relative activity was calculated and compared with the activity at 30°C (pH 7.4). The labels 1.1, 1.2 and 1.3 represent recombinant proteins r*Hc-*ACOX-1.1, -1.2 and -1.3, respectively.
(TIF)

S2 Fig Co-localisation of *Hc*-ACOX-1 and mitochondria in HEK293T cells. Green fluorescent protein (GFP)-fused *Hc*-ACOX-1 is expressed in HEK293T cells and the nuclei are stained with 4’,6-diamidino-2-phenylindole (DAPI). RFP/mitochondrion indicates red fluorescent protein expressed in mitochondrion. Scale bar: 10 μm.
(TIF)

S3 Fig Yeast two hybrid assay. *Saccharomyces cerevisiae* Y2HGold strain containing *Hc-*ACOX-1 mated with Y187 strain containing *Hc-*PEX-5 at 30°C for 24 h. (A-P) The mating strain was grown on SD/-Leu/-Trp (A-D, I-L) and SD/-Ade/-His/-Leu/-Trp (E-H, M-P) plates. AD-PEX5/BD-1.1 represents Y187 strain containing *Hc*-PEX-5 mating with Y2HGold strain containing *Hc*-ACOX-1.1. *Hc*-ACOX-1 without PTS1 is designated as *Hc*-ACOX-1 (-). (A) and (E), Negative control. (I) and (M), Positive control. Lam (lamin C) and p53 were used for negative control. T (T-antigen) and p53 were used for positive control. AD: activating domain expressed in Y187 strain; BD: binding domain expressed in Y2HGold strain.
(TIF)

S4 Fig Identification of anti-*Hc-*ACOX-1.1 polyclonal antibodies. (A-B) Identification of prepared polyclonal antibody using r*Hc*-ACOX-1 (A) and crude proteins from the fourth-stage larvae (L4s) of *Haemonchus contortus* (B) by western blot. (C) Incubation with pre-immune serum is performed as negative control.
(TIF)

S5 Fig Tissue immunolocalisation of *Hc*-ACOX-1 in the male adult worms of *Haemonchus contortus*. (A) Tissue distribution of *Hc*-ACOX-1 is indicated in the male adult worms of *H*. *contortus*, using anti-r*Hc*-ACOX-1.1 polyclonal antibodies and DAPI. (B) Pre-immune serum is used as the primary antibody in negative controls for the fourth-stage larvae (L4s), female (Af) and male (Am) adult worms of *H*. *contortus*. Fluorescein conjugated goat anti-rabbit IgG (H+L) is used as the secondary antibody. DIC: differential interference contrast, ct: cuticle, in: intestine, go: gonad, nu: nucleus, hd: hypodermis. Scale bar: 50 μm.
(TIF)

## References

[1] Michel JF. Arrested development of nematodes and some related phenomena. Adv Parasitol. 1974;12:279–366. doi: 10.1016/s0065-308x(08)60390-5 . 4281280

[2] Gibbs HC. Gastrointestinal nematodiasis in dairy cattle. J Dairy Sci. 1982;65(11):2182–8. doi: 10.3168/jds.S0022-0302(82)82480-6 . 6759538

[3] Gibbs HC. Hypobiosis in parasitic nematodes—an update. Adv Parasitol. 1986;25:129–74. 10.1016/S0065-308X(08)60343-7 . 3535434

[4] Golden JW, Riddle DL. The *Caenorhabditis elegans* dauer larva: developmental effects of pheromone, food, and temperature. Dev Biol. 1984;102(2):368–78. doi: 10.1016/0012-1606(84)90201-x . 6706004

[5] Cassada RC, Russell RL. The dauer larva, a post-embryonic developmental variant of the nematode *Caenorhabditis elegans*. Dev Biol. 1975;46(2):326–42. doi: 10.1016/0012-1606(75)90109-8 . 1183723

[6] Schad GA, Chowdhury AB, Dean CG, Kochar VK, Nawalinski TA, Thomas J, et al. Arrested development in human hookworm infections: an adaptation to a seasonally unfavorable external environment. Science. 1973;180(4085):502–4. doi: 10.1126/science.180.4085.502 . 17817813

[7] Sommerville RI, Davey KG. Diapause in parasitic nematodes: a review. Can J Zool. 2002;80(11):1817–40. Epub 2002/12/06. 10.1139/z02-163

[8] Haag ES, Fitch DH, Delattre M. From "the Worm" to "the Worms" and Back Again: The Evolutionary Developmental Biology of Nematodes. Genetics. 2018(2);210:397–433. Epub 2018/10/01. doi: 10.1534/genetics.118.300243 ; PubMed Central PMCID: PMC6216592. 30287515 PMC6216592

[9] Brenner S. The genetics of *Caenorhabditis elegans*. Genetics. 1974(1);77:71–94. ; PubMed Central PMCID: PMC1213120. 4366476 10.1093/genetics/77.1.71 PMC1213120

[10] von Reuss SH, Bose N, Srinivasan J, Yim JJ, Judkins JC, Sternberg PW, et al. Comparative metabolomics reveals biogenesis of ascarosides, a modular library of small-molecule signals in *C*. *elegans*. J Am Chem Soc. 2012;134(3):1817–24. Epub 2012/01/12. doi: 10.1021/ja210202y ; PubMed Central PMCID: PMC3269134. 22239548 PMC3269134

[11] Butcher RA, Ragains JR, Li W, Ruvkun G, Clardy J, Mak HY. Biosynthesis of the *Caenorhabditis elegans* dauer pheromone. Proc Natl Acad Sci U S A. 2009;106(6):1875–9. Epub 2009/01/27. doi: 10.1073/pnas.0810338106 ; PubMed Central PMCID: PMC 2631283. 19174521 PMC2631283

[12] Hu M, Lok JB, Ranjit N, Massey HC, Sternberg P W and Gasser R B. Structural and functional characterisation of the fork head transcription factor-encoding gene, *Hc-daf-16*, from the parasitic nematode *Haemonchus contortus* (Strongylida). Int J Parasitol. 2010;40(4):405–15. Epub 2009/09/29. doi: 10.1016/j.ijpara.2009.09.005 ; PubMed Central PMCID: PMC2853935. 19796644 PMC2853935

[13] Hand SC, Denlinger DL, Podrabsky JE, Roy R. Mechanisms of animal diapause: recent developments from nematodes, crustaceans, insects, and fish. Am J Physiol Regul Integr Comp Physiol. 2016;310(11):R1193–211. Epub 2016/04/06. doi: 10.1152/ajpregu.00250.2015 ; PubMed Central PMCID: PMC4935499. 27053646 PMC4935499

[14] Srinivasan J, Kaplan F, Ajredini R, Zachariah C, Alborn HT, Teal PE, et al. A blend of small molecules regulates both mating and development in *Caenorhabditis elegans*. Nature. 2008;454(7208):1115–8. Epub 2008/07/23. doi: 10.1038/nature07168 ; PubMed Central PMCID: PMC2774729. 18650807 PMC2774729

[15] Ludewig AH, Schroeder FC. Ascaroside signaling in *C*. *elegans*. WormBook. 2013;1–22. doi: 10.1895/wormbook.1.155.1 ; PubMed Central PMCID: PMC3758900. 23355522 PMC3758900

[16] Butcher RA, Fujita M, Schroeder FC, Clardy J. Small-molecule pheromones that control dauer development in *Caenorhabditis elegans*. Nat Chem Biol. 2007;3(7):420–2. Epub 2007/06/10. doi: 10.1038/nchembio.2007.3 . 17558398

[17] Pungaliya C, Srinivasan J, Fox BW, Malik RU, Ludewig AH, Sternberg PW, et al. A shortcut to identifying small molecule signals that regulate behavior and development in *Caenorhabditis elegans*. Proc Natl Acad Sci U S A. 2009;106(19):7708–13. Epub 2009/04/03. doi: 10.1073/pnas.0811918106 ; PubMed Central PMCID: PMC2683085. 19346493 PMC2683085

[18] Kim K, Sato K, Shibuya M, Zeiger DM, Butcher RA, Ragains JR, et al. Two chemoreceptors mediate developmental effects of dauer pheromone in *C*. *elegans*. Science. 2009;326(5955):994–8. Epub 2009/10/01. doi: 10.1126/science.1176331 ; PubMed Central PMCID: PMC4448937. 19797623 PMC4448937

[19] McGrath PT, Xu Y, Ailion M, Garrison JL, Butcher RA, Bargmann CI. Parallel evolution of domesticated Caenorhabditis species targets pheromone receptor genes. Nature. 2011;477(7364):321–5. Epub 2011/08/17. doi: 10.1038/nature10378 ; PubMed Central PMCID: PMC3257054. 21849976 PMC3257054

[20] Butcher RA, Ragains JR, Clardy J. An indole-containing dauer pheromone component with unusual dauer inhibitory activity at higher concentrations. Org Lett. 2009;11(14):3100–3. doi: 10.1021/ol901011c ; PubMed Central PMCID: PMC2726967. 19545143 PMC2726967

[21] Birnby DA, Link EM, Vowels JJ, Tian H, Colacurcio PL, Thomas JH. A transmembrane guanylyl cyclase (DAF-11) and Hsp90 (DAF-21) regulate a common set of chemosensory behaviors in *Caenorhabditis elegans*. Genetics. 2000;155(1):85–104. 10.1093/genetics/155.1.85 ; PubMed Central PMCID: PMC1461074. 10790386 PMC1461074

[22] Mutlu AS, Gao SM, Zhang H, Wang MC. Olfactory specificity regulates lipid metabolism through neuroendocrine signaling in *Caenorhabditis elegans*. Nat Commun. 2020;11(1):1450. Epub 2020/03/19. doi: 10.1038/s41467-020-15296-8; PubMed Central PMCID: PMC5453393. 32193370 PMC7081233

[23] Savage-Dunn C, Padgett RW. The TGF-β Family in *Caenorhabditis elegans*. Cold Spring Harb Perspect Biol. 2017;9(6):a022178. Epub 2017/06/01. doi: 10.1101/cshperspect.a022178; PubMed Central PMCID: PMC7081233. 28096268 PMC5453393

[24] Ren P, Lim CS, Johnsen R, Albert PS, Pilgrim D, Riddle DL. Control of *C*. *elegans* larval development by neuronal expression of a TGF-beta homolog. Science. 1996;274(5291):1389–91. doi: 10.1126/science.274.5291.1389 ; PubMed Central PMCID: PMC7081233. 8910282

[25] Pierce SB, Costa M, Wisotzkey R, Devadhar S, Homburger SA, Buchman AR, et al. Regulation of DAF-2 receptor signaling by human insulin and *ins-1*, a member of the unusually large and diverse *C*. *elegans* insulin gene family. Genes Dev. 2001;15(6):672–86. doi: 10.1101/gad.867301 ; PubMed Central PMCID: PMC312654. 11274053 PMC312654

[26] Li W, Kennedy SG, Ruvkun G. *daf-28* encodes a *C*. *elegans* insulin superfamily member that is regulated by environmental cues and acts in the DAF-2 signaling pathway. Genes Dev. 2003;17(7):844–58. Epub 2017/03/21. doi: 10.1101/gad.1066503 ; PubMed Central PMCID: PMC196030. 12654727 PMC196030

[27] Altintas O, Park S, Lee SJ. The role of insulin/IGF-1 signaling in the longevity of model invertebrates, *C*. *elegans* and *D*. *melanogaster*. BMB Rep. 2016;49(2):81–92. Epub 2016/02/28. doi: 10.5483/bmbrep.2016.49.2.261 ; PubMed Central PMCID: PMC4915121. 26698870 PMC4915121

[28] Mahanti P, Bose N, Bethke A, Judkins JC, Wollam J, Dumas KJ, et al. Comparative metabolomics reveals endogenous ligands of DAF-12, a nuclear hormone receptor, regulating *C*. *elegans* development and lifespan. Cell Metab. 2014;19(1):73–83. Epub 2015/01/07. doi: 10.1016/j.cmet.2013.11.024 ; PubMed Central PMCID: PMC3924769. 24411940 PMC3924769

[29] Antebi A, Yeh WH, Tait D, Hedgecock EM, Riddle DL. *daf-12* encodes a nuclear receptor that regulates the dauer diapause and developmental age in *C*. *elegans*. Genes Dev. 2000;14(12):1512–27. 10.1101/gad.14.12.1512 ; PubMed Central PMCID: PMC316684. 10859169 PMC316684

[30] Choe A, von Reuss SH, Kogan D, Gasser RB, Platzer EG, Schroeder FC, et al. Ascaroside signaling is widely conserved among nematodes. Curr Biol. 2012;22(9):772–80. Epub 2012/04/12. doi: 10.1016/j.cub.2012.03.024 ; PubMed Central PMCID: PMC3360977. 22503501 PMC3360977

[31] Crook M. The dauer hypothesis and the evolution of parasitism: 20 years on and still going strong. Int J Parasitol. 2014;44(1):1–8. Epub 2013/10/03. doi: 10.1016/j.ijpara.2013.08.004 ; PubMed Central PMCID: PMC3947200. 24095839 PMC3947200

[32] Noguez JH, Conner ES, Zhou Y, Ciche TA, Ragains JR, Butcher RA. A novel ascaroside controls the parasitic life cycle of the entomopathogenic nematode *Heterorhabditis bacteriophora*. ACS Chem Biol. 2012;7(6):961–6. Epub 2012/04/13. doi: 10.1021/cb300056q ; PubMed Central PMCID: PMC3548670. 22444073 PMC3548670

[33] Joo HJ, Yim YH, Jeong PY, Jin YX, Lee JE, Kim H, et al. *Caenorhabditis elegans* utilizes dauer pheromone biosynthesis to dispose of toxic peroxisomal fatty acids for cellular homoeostasis. Biochem J. 2009;422(1):61–71. Epub 2009/06/04. doi: 10.1042/BJ20090513 . 19496754

[34] Joo HJ, Kim KY, Yim YH, Jin YX, Kim H, Kim MY, et al. Contribution of the peroxisomal acox gene to the dynamic balance of daumone production in *Caenorhabditis elegans*. J Biol Chem. 2010;285(38):29319–25. Epub 2010/07/07. doi: 10.1074/jbc.M110.122663 ; PubMed Central PMCID: PMC2937964. 20610393 PMC2937964

[35] Zhang X, Feng L, Chinta S, Singh P, Wang Y, Nunnery J K, et al. Acyl-CoA oxidase complexes control the chemical message produced by *Caenorhabditis elegans*. Proc Natl Acad Sci U S A. 2015;112(13):3955–60. Epub 2015/03/16. doi: 10.1073/pnas.1423951112 ; PubMed Central PMCID: PMC4386371. 25775534 PMC4386371

[36] Tokuoka K, Nakajima Y, Hirotsu K, Miyahara I, Nishina Y, Shiga K, et al. Three-dimensional structure of rat-liver acyl-CoA oxidase in complex with a fatty acid: insights into substrate-recognition and reactivity toward molecular oxygen. J Biochem. 2006;139(4):789–95. Epub 2006/04/01. doi: 10.1093/jb/mvj088 . 16672280

[37] Ding H, Shi H, Shi Y, Guo X, Zheng X, Chen X, et al. Characterization and function analysis of a novel gene, *Hc-maoc-1*, in the parasitic nematode *Haemonochus contortus*. Parasit Vectors. 2017;10(1):67. Epub 2017/02/06. doi: 10.1186/s13071-017-1991-1; PubMed Central PMCID: PMC5294872. 28166831 PMC5294872

[38] Huang Y, Zheng X, Zhang H, Ding H, Guo X, Yang Y, et al. Site-Directed Mutagenesis Study Revealed Three Important Residues in Hc-DAF-22, a Key Enzyme Regulating Diapause of *Haemonchus contortus*. Front Microbiol. 2017;8:2176. Epub 2017/10/08. doi: 10.3389/fmicb.2017.02176; PubMed Central PMCID: PMC5682392. 29167662 PMC5682392

[39] Guo X, Zhang H, Zheng X, Zhou Q, Yang Y, Chen X, et al. Structural and functional characterization of a novel gene, *Hc-daf-22*, from the strongylid nematode *Haemonchus contortus*. Parasit Vectors. 2016;9(1):422. Epub 2016/07/29. doi: 10.1186/s13071-016-1704-1; PubMed Central PMCID: PMC4966567. 27472920 PMC4966567

[40] Yang Y, Guo X, Chen X, Zhou J, Wu F, Huang Y, et al. Functional characterization of a novel gene, *Hc-dhs-28* and its role in protecting the host after *Haemonchus contortus* infection through regulation of diapause formation. Int J Parasitol. 2020;50(12):945–957. Epub 2020/08/25. doi: 10.1016/j.ijpara.2020.04.013 . 32858035

[41] Soulsby EJ. Helminths, Arthropods and Protozoa of Domesticated Animals. 7th ed. Longstaffe JA, editor. Baillière Tindall (London): Transactions of The Royal Society of Tropical Medicine and Hygiene; 1984.

[42] Schallig HD, Hornok S, Cornelissen JB. Comparison of two enzyme immunoassays for the detection of *Haemonchus contortus* infections in sheep. Vet Parasitol. 1995;57(4):329–38. Epub 2000/04/05. doi: 10.1016/0304-4017(94)00693-7 . 7660570

[43] Blitz NM, Gibbs HC. Morphological characterizatioon of the stage of arrested development of *Haemonchus contortus* in sheep. Can J Zool. 1971;47(7):991–5. doi: 10.1139/z71-151 . 5118671

[44] Golden JW, Riddle DL. A pheromone influences larval development in the nematode *Caenorhabditis elegans*. Science. 1982;218(4572):578–80. doi: 10.1126/science.6896933 . 6896933

[45] Jeong PY, Jung M, Yim YH, Kim H, Park M, Hong E, et al. Chemical structure and biological activity of the *Caenorhabditis elegans* dauer-inducing pheromone. Nature. 2005;433(7025):541–5. doi: 10.1038/nature03201 . 15690045

[46] Guo H, La Clair JJ, Masler EP, O’Doherty G, Xing Y. De Novo Asymmetric Synthesis and Biological Analysis of the Daumone Pheromones in *Caenorhabditis elegans* and in the Soybean Cyst Nematode *Heterodera glycines*. Tetrahedron. 2016;72(18):2280–6. Epub 2016/03/16. doi: 10.1016/j.tet.2016.03.033 ; PubMed Central PMCID: PMC5809136. 29445247 PMC5809136

[47] Choe A, Chuman T, von Reuss SH, Dossey AT, Yim JJ, Ajredini R, et al. Sex-specific mating pheromones in the nematode *Panagrellus redivivus*. Proc Natl Acad Sci U S A. 2012;109(51):20949–54. Epub 2012/10/03. doi: 10.1073/pnas.1218302109 ; PubMed Central PMCID: PMC3529029. 23213209 PMC3529029

[48] Rachubinski RA, Subramani S. How proteins penetrate peroxisomes. Cell.1995;83(4):525–8. Epub 2004/04/14. doi: 10.1016/0092-8674(95)90091-8 . 7585954

[49] Bottger G, Barnett P, Klein AT, Kragt A, Tabak HF, Distel B. *Saccharomyces cerevisiae* PTS1 receptor Pex5p interacts with the SH3 domain of the peroxisomal membrane protein Pex13p in an unconventional, non-PXXP-related manner. Mol Biol Cell. 2000;11(11):3963–76. Epub 2017/10/13. doi: 10.1091/mbc.11.11.3963 ; PubMed Central PMCID: PMC15050. 11071920 PMC15050

[50] Dmochowska A, Dignard D, Maleszka R, Thomas DY. Structure and transcriptional control of the *Saccharomyces cerevisiae* POX1 gene encoding acyl-coenzyme A oxidase. Gene. 1990;88(2):247–52. Epub 2003/01/29. doi: 10.1016/0378-1119(90)90038-s . 2189786

[51] Subramani S. Components involved in peroxisome import, biogenesis, proliferation, turnover, and movement. Physiol Rev. 1998;78(1):171–88. Epub 1998/01/01. doi: 10.1152/physrev.1998.78.1.171 . 9457172

[52] Distel B, Erdmann R, Gould SJ, Blobel G, Crane DI, Cregg JM, et al, A unified nomenclature for peroxisome biogenesis factors. J Cell Biol. 1996;135(1):1–3. Epub 1996/10/01. doi: 10.1083/jcb.135.1.1 ; PubMed Central PMCID: PMC2121017. 8858157 PMC2121017

[53] Lazarow PB, Fujiki Y. Biogenesis of peroxisomes. Annu Rev Cell Biol. 1985;1:489–530. doi: 10.1146/annurev.cb.01.110185.002421 . 3916321

[54] Agne B, Meindl NM, Niederhoff K, Einwachter H, Rehling P, Sickmann A, et al. Pex8p: an intraperioxisomal organizer of the peroxisomal import machinery. Mol Cell. 2003;11(3):635–646. Epub 2004/04/16. doi: 10.1016/s1097-2765(03)00062-5 . 12667447

[55] Rayapuram N, Subramani S. The importomer-a peroxisomal membrane complex involved in protein translocation into the peroxisome matrix. Biochim Biophys Acta. 2006;1763(12):1613–19. Epub 2006/08/30. doi: 10.1016/j.bbamcr.2006.08.035 . 17027097

[56] Brocard C, Kragler F, Simon MM, Schuster T, Hartig A. The tetratricopeptide repeat-domain of the PAS10 protein of *Saccharomyces* cerevisiae is essential for binding the peroxisomal targeting signal-SKL. Biochem Biophys Res. 1994;204(3):1016–22. Epub 2002/05/25. doi: 10.1006/bbrc.1994.2564 . 7980572

[57] Terlecky SR, Nuttley WM, McCollum D, Sock E, Subramani S. The *Pichia pastoris* peroxisomal protein PAS8p is the receptor for the C-terminal tripeptide peroxisomal targeting signal. EMBO J. 1995;14(15):3627–34. 10.1002/j.1460-2075.1995.tb00032.x ; PubMed Central PMCID: PMC394437. 7641682 PMC394437

[58] Nikolaou S, Gasser RB. Extending from PARs in *Caenorhabditis elegans* to homologues in *Haemonchus contortus* and other parasitic nematodes. Parasitology. 2007;134(Pt4):461–82. Epub 2006/10/16. doi: 10.1017/S0031182006001727 . 17107637

[59] Zhou Y, Wang Y, Zhang X, Bhar S, Jones Lipinski RA, Han J, et al. Biosynthetic tailoring of existing ascaroside pheromones alters their biological function in *C*. *elegans*. Elife. 2018;7: e33286. doi: 10.7554/eLife.33286; PubMed Central PMCID: PMC5986272. 29863473 PMC5986272

[60] Yang Y, Guo X, Zhang H, Huang Y, Chen X, Du A. Characterization of the development of *Haemonchus contortus* ZJ strain from gerbils. Parasit Vectors. 2017;10(1):505. doi: 10.1186/s13071-017-2465-1; PubMed Central PMCID: PMC5651610. 29058609 PMC5651610

[61] Cox DD, Todd AC. Survey of gastrointestinal parasitism in Wisconsin dairy cattle. J Am Vet Med Assoc. 1962;141:706–9. . 13881890

[62] Schwarz EM, Korhonen PK, Campbell BE, Young ND, Jex AR, Jabbar A, et al. The genome and developmental transcriptome of the strongylid nematode *Haemonchus contortus*. Genome Biol. 2013;14(8):R89. Epub 2013/08/28. doi: 10.1186/gb-2013-14-8-r89; PubMed Central PMCID: PMC4053716. 23985341 PMC4053716

[63] Doyle SR, Tracey A, Laing R, Holroyd N, Bartley D, Bazant W, et al. Genomic and transcriptomic variation defines the chromosome-scale assembly of *Haemonchus contortus*, a model gastrointestinal worm. Commun Biol. 2020;3(1):656. Epub 2020/10/09. doi: 10.1038/s42003-020-01377-3; PubMed Central PMCID: PMC7652881. 33168940 PMC7652881

[64] Serroni A, Magistrali CF, Pezzotti G, Bano L, Pellegrini M, Severi G, et al. Expression of deleted, atoxic atypical recombinant beta2 toxin in a baculovirus system and production of polyclonal and monoclonal antibodies. Microb Cell Fact. 2017;16(1):94. Epub 2017/05/27. doi: 10.1186/s12934-017-0707-8; PubMed Central PMCID: PMC5445335. 28545467 PMC5445335

[65] Zhang L, Mou L, Chen X, Yang Y, Hu M, Li X, et al. Identification and preliminary characterization of *Hc-clec-160*, a novel C-type lectin domain-containing gene of the strongylid nematode *Haemonchus contortus*. Parasit Vectors. 2018;11(1):430. Epub 2018/07/20. doi: 10.1186/s13071-018-3005-3; PubMed Central PMCID: PMC6054721. 30029661 PMC6054721

[66] Yang Y, Zhang G, Wu J, Chen X, Tong D, Yang Y, et al. Recombinant HcGAPDH Protein Expressed on Probiotic *Bacillus subtilis* Spores Protects Sheep from *Haemonchus contortus* Infection by Inducing both Humoral and Cell-Mediated Responses. mSystems. 2020;5(3):e00239–20. Epub 2020/05/12. doi: 10.1128/mSystems.00239-20 ; PubMed Central PMCID: PMC7219552. 32398277 PMC7219552

[67] Geldhof P, Murray L, Couthier A, Gilleard JS, McLauchlan G, Knox D P, et al. Testing the efficacy of RNA interference in *Haemonchus contortus*. Int J Parasitol. 2006;36(7):801–10. Epub 2006/01/18. doi: 10.1016/j.ijpara.2005.12.004 . 16469321

