# Supplementary material for: Acyl-CoA oxidase ACOX-1 interacts with a peroxin PEX-5 to play roles in larval development of Haemonchus contortus
Source: PLoS Pathog. 2021 Jul 16;17(7):e1009767. doi: 10.1371/journal.ppat.1009767 (PMC8354476; doi:10.1371/journal.ppat.1009767)
Supplement: S1 Table — (DOCX) [file ppat.1009767.s001.docx]

S1Table List of primers used in this study.

| **Primer** | **Purpose** | **Sequence(5’-3’)** |
| --- | --- | --- |
| *Hc-acox-1.1* 1F | cDNA cloning | ATGCCTTTGAATAAACTACTTCGCGA |
| *Hc-acox-1.1* 1R | cDNA cloning | TCACAATTTGCTCTGAGCATGGG |
| *Hc-acox-1.2* 1F | cDNA cloning | ATGCCTTTTAGAGGAATTACCGTCG |
| *Hc-acox-1.2* 1R | cDNA cloning | TTACAGCTTGCTTTGCTCCCTAGC |
| *Hc-acox-1.3* 1F | cDNA cloning | ATGCCTTTTAGAGGAATTACCGTCG |
| *Hc-acox-1.3* 1R | cDNA cloning | TTACAGCTTGCTTTGCTCCCTAGCC |
| *Ce-acox-1a* 1F | cDNA cloning | ATGGTGCATCTCAACAAAACAATTC |
| *Ce-acox-1a* 1R | cDNA cloning | TTATAGCTTGCTCTGCCTGGCT |
| *Ce-acox-1b* 1F | cDNA cloning | ATGGCTCTTCACGGTGTGATGTC |
| *Ce-acox-1b* 1R | cDNA cloning | TTATAGCTTGCTCTGCCTGGCTTTT |
| *Ce-acox-1c* 1F | cDNA cloning | ATGGCTGCACAGATTTATGGGG |
| *Ce-acox-1c* 1R | cDNA cloning | TTATAGCTTGCTCTGCCTGGCTTTT |
| *Hc-acox-1.1* 2F | Prokaryotic expression | CTCGAGGGATCCGAATTCATGCCTTTGAATAAACTACTTC |
| *Hc-acox-1.1* 2R | Prokaryotic expression | GATTACCTATCTAGACTGCAGTTAATGATGATGATGATGATGCTGAGCATGGGCTTCCATCATCATT |
| *Hc-acox-1.2* 2F | Prokaryotic expression | CTCGAGGGATCCGAATTCATGCCTTTTAGAGGAATTACC |
| *Hc-acox-1.2* 2R | Prokaryotic expression | GATTACCTATCTAGACTGCAGTTAATGATGATGATGATGATGAATGATGAAGCAAGCTAGGGAGCAA |
| *Hc-acox-1.3* 2F | Prokaryotic expression | CCCTCGAGGGATCCGAATTCATGCCTTTTAGAGGAATTAC |
| *Hc-acox-1.3* 2R | Prokaryotic expression | GATTACCTATCTAGACTGCAGTTAATGATGATGATGATGATGAATGATGAAGCAGGCTAGGGAGCAA |
| *Hc-acox-1.1* 3F | RT-PCR | TGGAGTGAACCCAACTAACGC |
| *Hc-acox-1.1* 3R | RT-PCR | GTCCGTACAGCCCGATGACT |
| *Hc-acox-1.2* 3F | RT-PCR | ATGTCGGAACTTTATGGTGTGG |
| *Hc-acox-1.2* 3R | RT-PCR | TATTCTCTCCTTCGTATGTAGCAC |
| *Hc-acox-1.3* 3F | RT-PCR | GTCAAGGTTACCCCCGAGG |
| *Hc-acox-1.3* 3R | RT-PCR | AGTAGCCGACTTTAGCGTGAG |
| *Hc-β-tubulin* F | RT-PCR | GTTGGTAACTCGACTGCTATCC |
| *Hc-β-tubulin* R | RT-PCR | CTCCATTTCGTCCATACCCTC |
| *Hc-acox-1.1* 4F | Subcellular localization | TCAGATCTCGAGCTCAAGCTTCATGCCTTTGAATAAACTACTTC  GCGA |
| *Hc-acox-1.1* 4R | Subcellular localization | TTATCTAGATCCGGTGGATCCTCACAATTTGCTCTGAGCATGGG |
| *Hc-acox-1.2* 4F | Subcellular localization | GATCTCGAGCTCAAGCTTCATGCCTTTTAGAGGAATTACCG TCG |
| *Hc-acox-1.2* 4R | Subcellular localization | TTATCTAGATCCGGTGGATCCTTACAGCTTGCTTTGCTCCCTAGC |
| *Hc-acox-1.3* 4F | Subcellular localization | GATCTCGAGCTCAAGCTTCATGCCTTTTAGAGGAATTACCG TCG |
| *Hc-acox-1.3* 4R | Subcellular localization | TATCTAGATCCGGTGGATCCTTACAGCTTGCTTTGCTCCCTAGC C |
| *Hc-acox-1.1* 5R | Subcellular localization (ΔPTS1) | TATCTAGATCCGGTGGATCCTTACTGAGCATGGGCTTCCATCAT C |
| *Hc-acox-1.2* 5R | Subcellular localization (ΔPTS1) | TTATCTAGATCCGGTGGATCCTTATTGCTCCCTAGCTTGCTTCATC  AT |
| *Hc-acox-1.3* 5R | Subcellular localization (ΔPTS1) | TATCTAGATCCGGTGGATCCTTATTGCTCCCTAGCCTGCTTCATC |
| *Hc-acox-1.1* 6F | Enzyme activity  (first cycle) | ATAAGGACTATAAGGACGATGATGACAAGGACTACAAAGATGAT  GACGATAAACCTTTGAATAAACTACTTCGCGATGG |
| *Hc-acox-1.1* 6R | Enzyme activity  (two cycles) | CCACACTGGACTAGTGGATCCTCACAATTTGCTCTGAGCATGGG |
| *Hc-acox-1.2/3* 6F | Enzyme activity  (first cycle) | ATAAGGACTATAAGGACGATGATGACAAGGACTACAAAGATGAT  GACGATAAACCTTTTAGAGGAATTACCGTCGGTG |
| *Hc-acox-1.2/3* 6R | Enzyme activity  (two cycles) | CACACTGGACTAGTGGATCCTTACAGCTTGCTTTGCTCCCTAGC |
| *Hc-acox-1.1* 6F | Enzyme activity  (second cycle) | CTAGCGTTTAAACTTAAGCTTCGCCACCATGGATTACAAGGATG  ACGACGATAAGGACTATAAGGACGATGATGACAA |
| *Hc-acox-1.1* 7F | Spotting assay | CATCACCATCACCATGCTAGCCAAAATGCATCATCATCATCATCA  CATGCCTTTGAATAAACTACTTCGCGA |
| *Hc-acox-1.1* 7R | Spotting assay | AGAAGTCCAAAGCTGGATCCTCACAATTTGCTCTGAGCATGGG |
| *Hc-acox-1.2/3* 7F | Spotting assay | ATCACCATCACCATGCTAGCCAAAATGCATCATCATCATCATCAC  ATGCCTTTTAGAGGAATTACCGTCG |
| *Hc-acox-1.2/3* 7R | Spotting assay | AGAAGTCCAAAGCTGGATCCTTACAGCTTGCTTTGCTCCCTAGC |
| *Hc-acox-1.1* 8R | Spotting assay (ΔPTS1) | GAAGTCCAAAGCTGGATCCTCACTGAGCATGGGCTTCCATCATC |
| *Hc-acox-1.2/3* 8R | Spotting assay (ΔPTS1) | AAGTCCAAAGCTGGATCCTTATTGCTCCCTAGCTTGCTTCATCAT |
| *Hc-acox-1.1* 9F | Spotting assay | TCACCATCACCATGCTAGCCAAAATGCATCATCATCATCATCACA |
| *Hc-acox-1.1* 10R | Spotting assay (T151A) | GGCCTAATTCTGCTTGGGCATAGGTTCCGATGATCT |
| *Hc-acox-1.1* 10F | Spotting assay (T151A) | CTATGCCCAAGCAGAATTAGGCCACGGTACAAATCTGAG |
| *Hc-acox-1.1* 11R | Spotting assay (G190A) | TGCCAAGATTTGCAGGCCACCATTTCATCGCTGT |
| *Hc-acox-1.1* 11F | Spotting assay (G190A) | ATGGTGGCCTGCAAATCTTGGCAAAAGTGCAAATCATG |
| *Hc-acox-1.1* 12R | Spotting assay (E433A) | TATCAAAGGCTGCCAGATGGGCTTTGTAATCGTAATCAA |
| *Hc-acox-1.1* 12F | Spotting assay (E433A) | AGCCCATCTGGCAGCCTTTGATAAGGCAGCAAAACTC |
| *Hc-acox-1.2* 9R | Spotting assay (E206A) | TATTCTCTCCTGCGTATGTAGCACCGCCAATGGCC |
| *Hc-acox-1.2* 9F | Spotting assay (E206A) | TGCTACATACGCAGGAGAGAATATGGTCATGCTCCAACA |
| *Hc-acox-1.3* 9R | Spotting assay (E206A) | TATTCTCTCCTGCATATGTAGCACCACCAATGGCCA |
| *Hc-acox-1.3* 9F | Spotting assay (E206A) | TGCTACATATGCAAGGAGAGAATATGGTCATGCTCCAGC |
| *Hc-acox-1.1* 13F | Y2H | GAGGAGGACCTGCATATGATGCCTTTGAATAAACTACTTCGCGA |
| *Hc-acox-1.1*13R | Y2H | CGCTGCAGGTCGACGGATCCTCACAATTTGCTCTGAGCATGGG |
| *Hc-acox-1.2/3* 10F | Y2H | GAGGAGGACCTGCATATGATGCCTTTTAGAGGAATTACCGTCG |
| *Hc-acox-1.2/3* 10R | Y2H | GCTGCAGGTCGACGGATCCTTACAGCTTGCTTTGCTCCCTAGC |
| *Hc-acox-1.1* 14R | Y2H (ΔPTS1) | CTGCAGGTCGACGGATCCTCACTGAGCATGGGCTTCCATCATC |
| *Hc-acox-1.2* 11R | Y2H (ΔPTS1) | CTGCAGGTCGACGGATCCTTATTGCTCCCTAGCTTGCTTCATCAT |
| *Hc-acox-1.3* 11R | Y2H (ΔPTS1) | GCTGCAGGTCGACGGATCCTTATTGCTCCCTAGCCTGCTTCATC |
| *Hc-pex-1* 1F | cDNA cloning | ATGTCTAAAAGTGCGGTCCCTATTCTG |
| *Hc-pex-1* 1R | cDNA cloning | CGACAGAGCACCTTCTCAAAGCAT |
| *Hc-pex-3* 1F | cDNA cloning | ATGATCCTGACACTTGAGGCGTTTA |
| *Hc-pex-3* 1R | cDNA cloning | CTAGGAATTGAAAACGTAAACGGCA |
| *Hc-pex-5* 1F | cDNA cloning | ATGAAGTCGGTGGTGGAAGCC |
| *Hc-pex-5* 1R | cDNA cloning | TCATTCAGTTCCCATTCTAGCTAGCTC |
| *Hc-pex-19* 1F | cDNA cloning | ATGTCTGGTAAGGATGATGGTTCTTCA |
| *Hc-pex-19* 1R | cDNA cloning | TTACATCAGTGTACAGGCATTCGCA |
| *Ce-pex-5* 1F | cDNA cloning | ATGAAAGGAGTTGTAGAAGGACAATGTG |
| *Ce-pex-5* 1R | cDNA cloning | CTAGACTAGAGAGGCTTTTACAGCTGCC |
| *Hc-pex-1* 2F | Y2H | TGGAGGCCAGTGAATTCATGTCTAAAAGTGCGGTCCCTATTCTG |
| *Hc-pex-1* 2R | Y2H | GCTCGAGCTCGATGGATCCCGACAGAGCACCTTCTCAAAGCAT |
| *Hc-pex-3* 2F | Y2H | CATGGAGGCCAGTGAATTCATGATCCTGACACTTGAGGCGTTTA |
| *Hc-pex-3* 2R | Y2H | CTCGAGCTCGATGGATCCCTAGGAATTGAAAACGTAAACGGCA |
| *Hc-pex-5* 2F | Y2H | TGGAGGCCAGTGAATTCATGTCTGGTAAGGATGATGGTTCTTCA |
| *Hc-pex-5* 2R | Y2H | GCTCGAGCTCGATGGATCCTTACATCAGTGTACAGGCATTCGCA |
| *Hc-pex-19* 2F | Y2H | TGGAGGCCAGTGAATTCATGTCTGGTAAGGATGATGGTTCTTCA |
| *Hc-pex-19* 2R | Y2H | GCTCGAGCTCGATGGATCCTTACATCAGTGTACAGGCATTCGCA |
| *Hc-acox-1.1* 15R | CoIP (ΔPTS1) | ACACTGGACTAGTGGATCCTCACTGAGCATGGGCTTCCATCATC |
| *Hc-acox-1.2/3* 12R | CoIP (ΔPTS1) | CACTGGACTAGTGGATCCTTATTGCTCCCTAGCTTGCTTCATCAT |
| *Hc-pex-1* 3F | CoIP (first cycle) | GGCTATCCCTATGATGTGCCCGATTATGCGTATCCTTACGATGTTC  CAGATTATGCCATGTCTAAAAGTGCGGTCCCTATTCTG |
| *Hc-pex-1* 3R | CoIP (two cycles) | TGGACTAGTGGATCCTTAGACGACAGAGCACCTTCTCAAAGCA |
| *Hc-pex-3* 3F | CoIP (first cycle) | GGCTATCCCTATGATGTGCCCGATTATGCGTATCCTTACGATGTTC  CAGATTATGCCATGTCAGGCGCGTGGGAATT |
| *Hc-pex-3* 3R | CoIP (two cycles) | TGGACTAGTGGATCCCTATTTATACTGAGCAATAAGTGCACTGCC |
| *Hc-pex-5* 3F | CoIP (first cycle) | GGCTATCCCTATGATGTGCCCGATTATGCGTATCCTTACGATGTTC  CAGATTATGCCATGAAGTCGGTGGTGGAAGCC |
| *Hc-pex-5* 3R | CoIP (two cycles) | CACTGGACTAGTGGATCCTCATTCAGTTCCCATTCTAGCTAGCTC |
| Hc-pex-19 3F | CoIP (first cycle) | GGCTATCCCTATGATGTGCCCGATTATGCGTATCCTTACGATGTTC  CAGATTATGCCATGTCTGGTAAGGATGATGGTTCTTCA |
| Hc-pex-19 3R | CoIP (two cycles) | ACACTGGACTAGTGGATCCTTACATCAGTGTACAGGCATTCGCG |
| *Hc-pex-1* (3,5,19) 4F | CoIP (second cycle) | CTAGCGTTTAAACTTAAGCTTCGCCACCATGGAATACCCGTACG  ACGTCCCGGACTACGCTGGCTATCCCTATGATGTGCCCG |
| *Hc-acox-1.1* 16F | RNAi | TGGATCCCCCGGGCTGCAGATGCCTTTGAATAAACTACTTCGCG |
| *Hc-acox-1.1* 16R | RNAi | TCGACGGTATCGATAAGCTTCATGTTGGTCCTTGGAATTCGGTA |
| *Hc-acox-1.2/3* 13F | RNAi | TGGATCCCCCGGGCTGCAGATGCCTTTTAGAGGAATTACCGTCG |
| *Hc-acox-1.2/3* 13R | RNAi | TCGACGGTATCGATAAGCTTTTACAGCTTGCTTTGCTCCCTAGC |
| *Hc-tropomyosin* 1F | RNAi | TGGATCCCCCGGGCTGCAGATGGACGCCATCAAGAAGAAGATG |
| *Hc-tropomyosin* 1R | RNAi | GACGGTATCGATAAGCTTTTAGACACCATTAAGTTCGGTGAGCA |
| *At-Lhcb-4.3* 1F | RNAi | CGTGGATCCCCCGGGCTGCAGACTCAATATGGCTACCACCACGC |
| *At-Lhcb-4.3* 1R | RNAi | CGACGGTATCGATAAGCTTCCATATCAACGTCGTCAACGAGAAC |
| *Hc-tropomyosin* 2F | RT-PCR | AGGAGCTTGAACGTGCTGAA |
| *Hc-tropomyosin* 2R | RT-PCR | TCGGACTCGTCGACATTGTG |
| *At-Lhcb-4.3* 2F | RT-PCR | ACTCAATATGGCTACCACCACTGC |
| *At-Lhcb-4.3* 2R | RT-PCR | TTCCAGGTCTAGGATCTTGAATC |

RT-PCR: SYBR Green real-time PCR, ΔPTS1: deletion of peroxisomal targeting signal type 1 (SKL), Y2H: yeast two hybrid, CoIP: Co-immunoprecipitation, RNAi: RNA interference.
