# Supplementary figures and images for: Acyl-CoA oxidase ACOX-1 interacts with a peroxin PEX-5 to play roles in larval development of Haemonchus contortus
Source: PLoS Pathog. 2021 Jul 16;17(7):e1009767. doi: 10.1371/journal.ppat.1009767 (PMC8354476; doi:10.1371/journal.ppat.1009767)

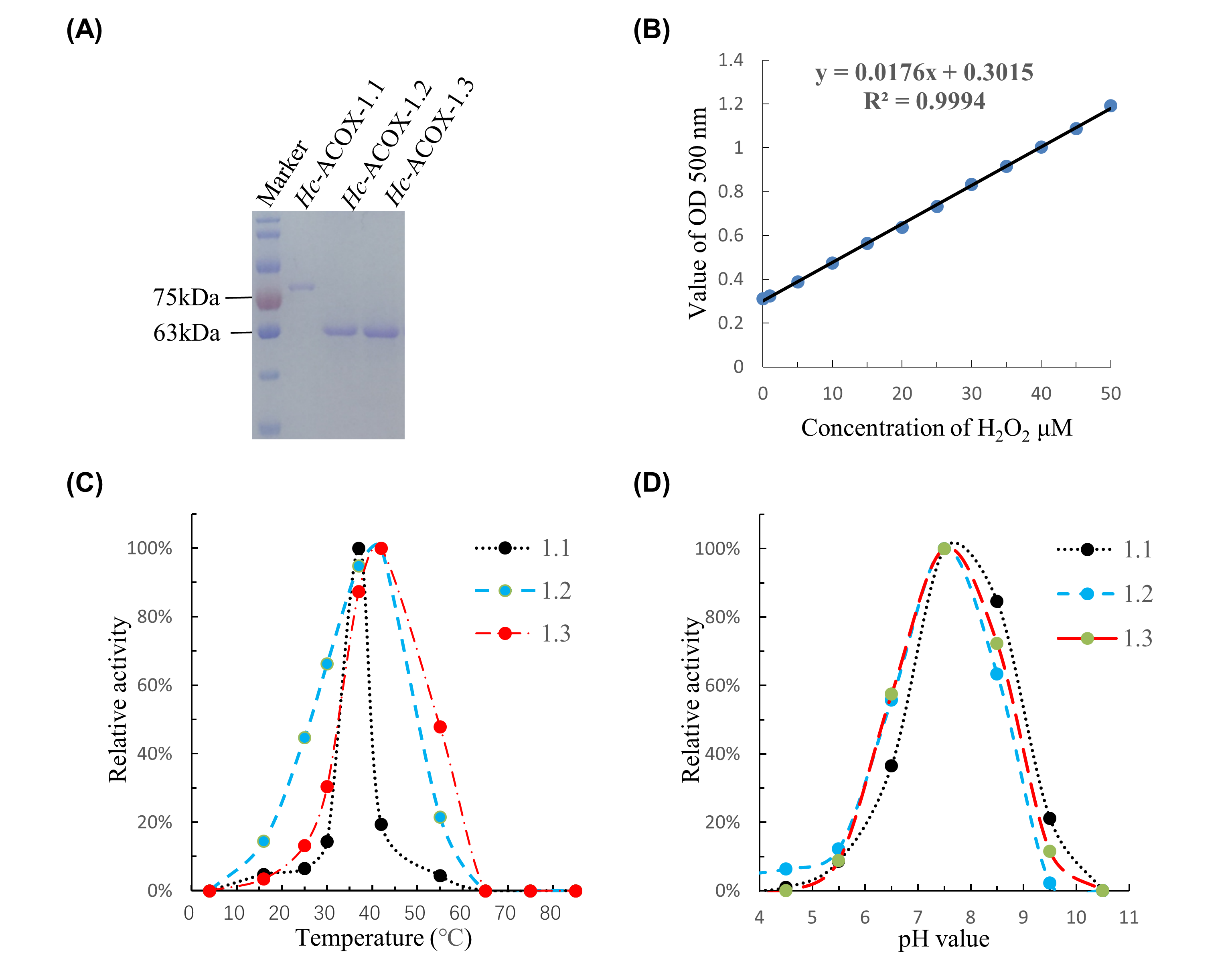

Supplement: S1 Fig — (A) Validation of eukaryotic expression of Hcdacox-1 in HEK293T cells for the purity using SDS-PAGE. (B) Construction of standard curve using hydrogen peroxide as substrate. (C-D) Determination of optimum temperature (C) at pH 7.4 and optimum pH value (D) at 30°C using palmitoyl-CoA as substrate. Relative activity was calculated and compared with the activity at 30°C (pH 7.4). The labels 1.1, 1.2 and 1.3 represent recombinant proteins rHc-ACOX-1.1, -1.2 and -1.3, respectively. (TIF) [file ppat.1009767.s002.tif]

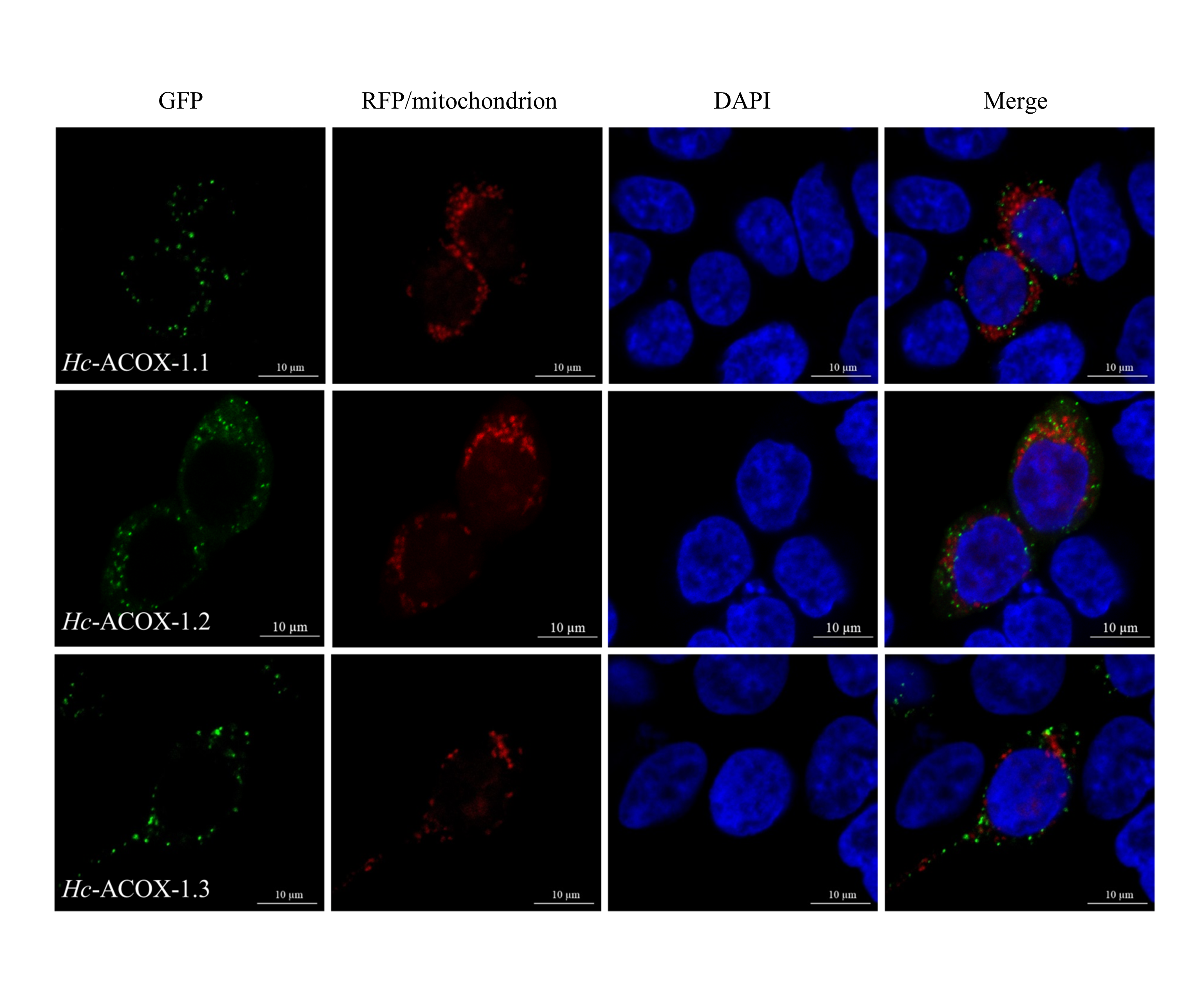

Supplement: S2 Fig — Green fluorescent protein (GFP)-fused Hc-ACOX-1 is expressed in HEK293T cells and the nuclei are stained with 4’,6-diamidino-2-phenylindole (DAPI). RFP/mitochondrion indicates red fluorescent protein expressed in mitochondrion. Scale bar: 10 μm. (TIF) [file ppat.1009767.s003.tif]

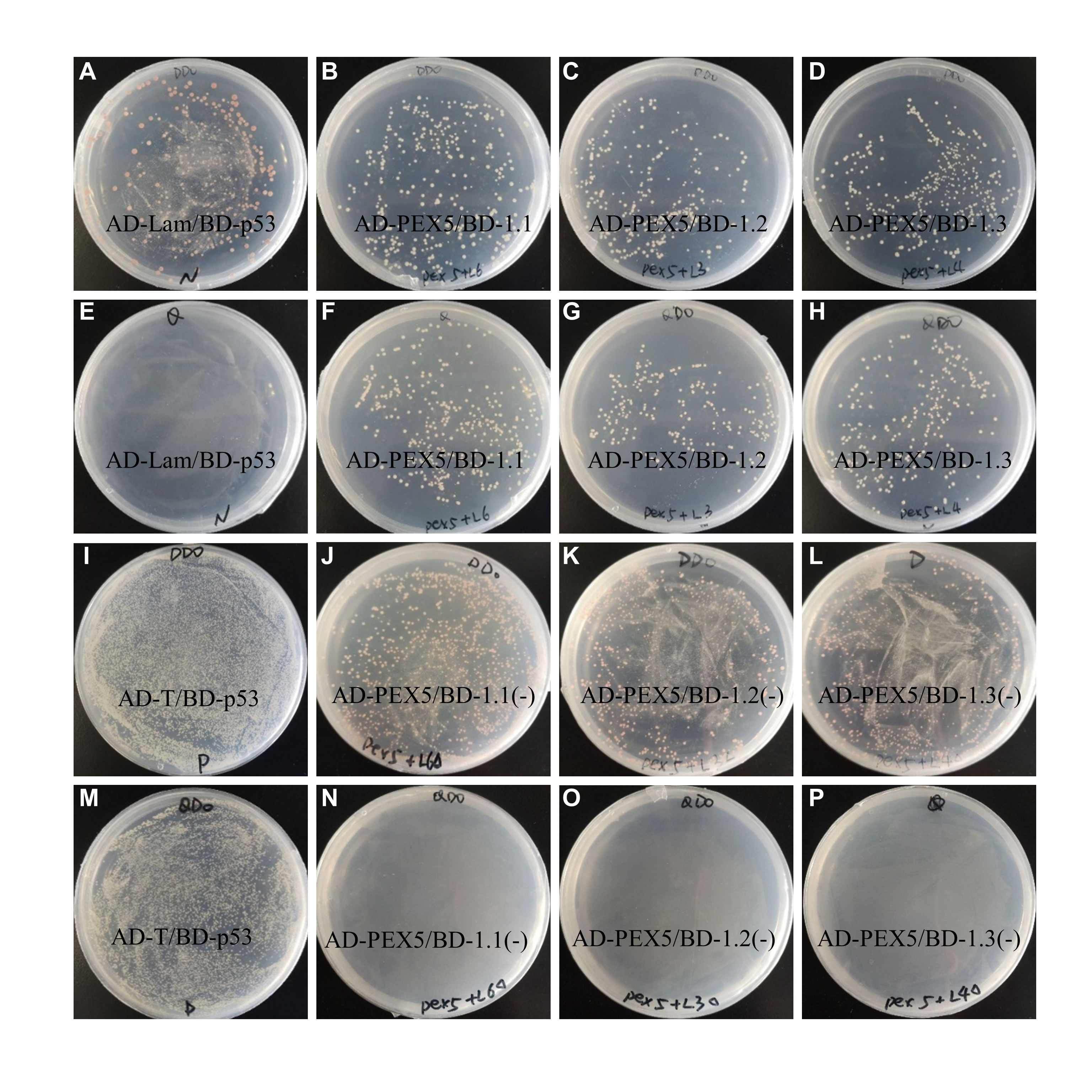

Supplement: S3 Fig — Saccharomyces cerevisiae Y2HGold strain containing Hc-ACOX-1 mated with Y187 strain containing Hc-PEX-5 at 30°C for 24 h. (A-P) The mating strain was grown on SD/-Leu/-Trp (A-D, I-L) and SD/-Ade/-His/-Leu/-Trp (E-H, M-P) plates. AD-PEX5/BD-1.1 represents Y187 strain containing Hc-PEX-5 mating with Y2HGold strain containing Hc-ACOX-1.1. Hc-ACOX-1 without PTS1 is designated as Hc-ACOX-1 (-). (A) and (E), Negative control. (I) and (M), Positive control. Lam (lamin C) and p53 were used for negative control. T (T-antigen) and p53 were used for positive control. AD: activating domain expressed in Y187 strain; BD: binding domain expressed in Y2HGold strain. (TIF) [file ppat.1009767.s004.tif]

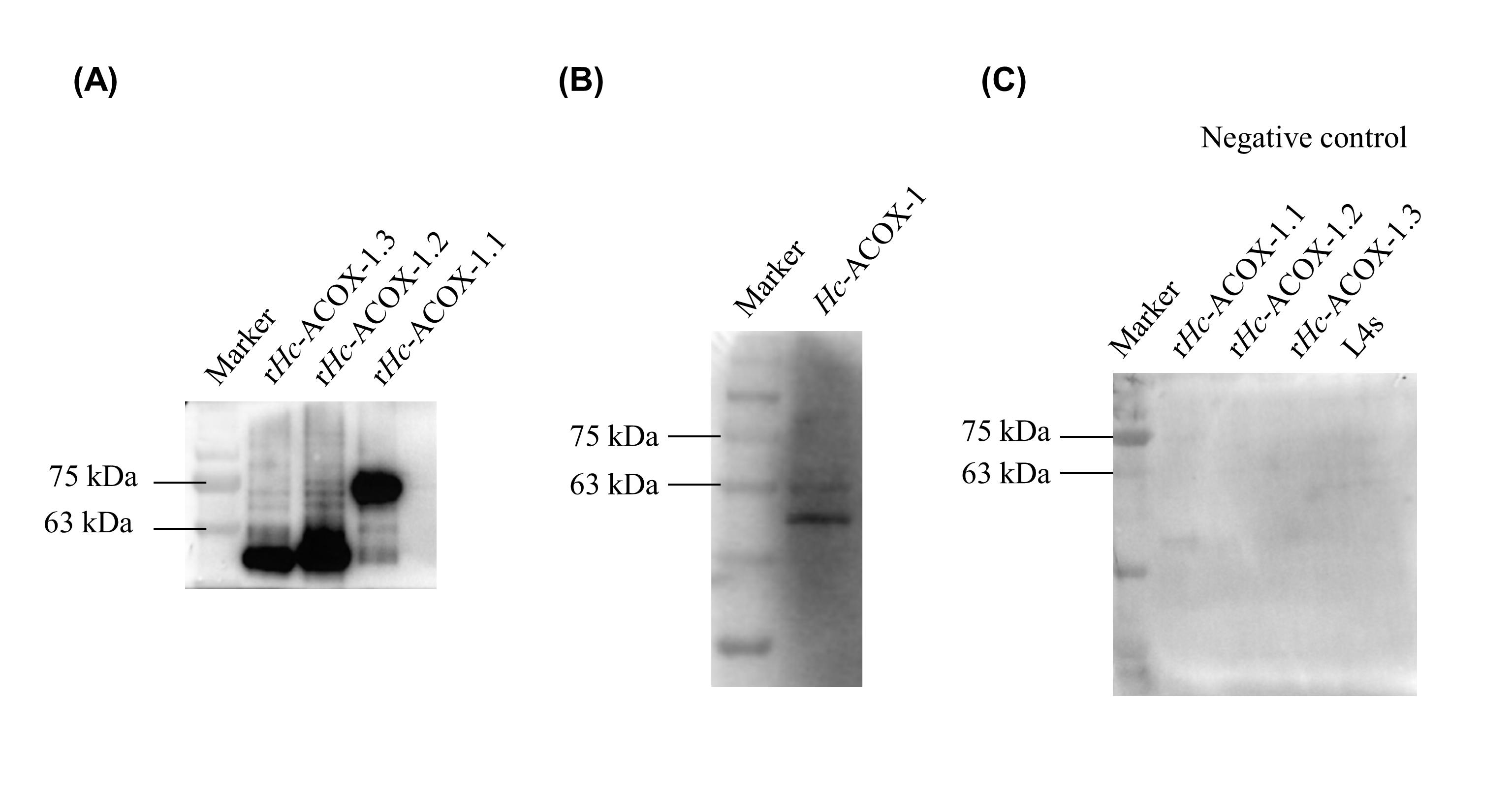

Supplement: S4 Fig — (A-B) Identification of prepared polyclonal antibody using rHc-ACOX-1 (A) and crude proteins from the fourth-stage larvae (L4s) of Haemonchus contortus (B) by western blot. (C) Incubation with pre-immune serum is performed as negative control. (TIF) [file ppat.1009767.s005.tif]

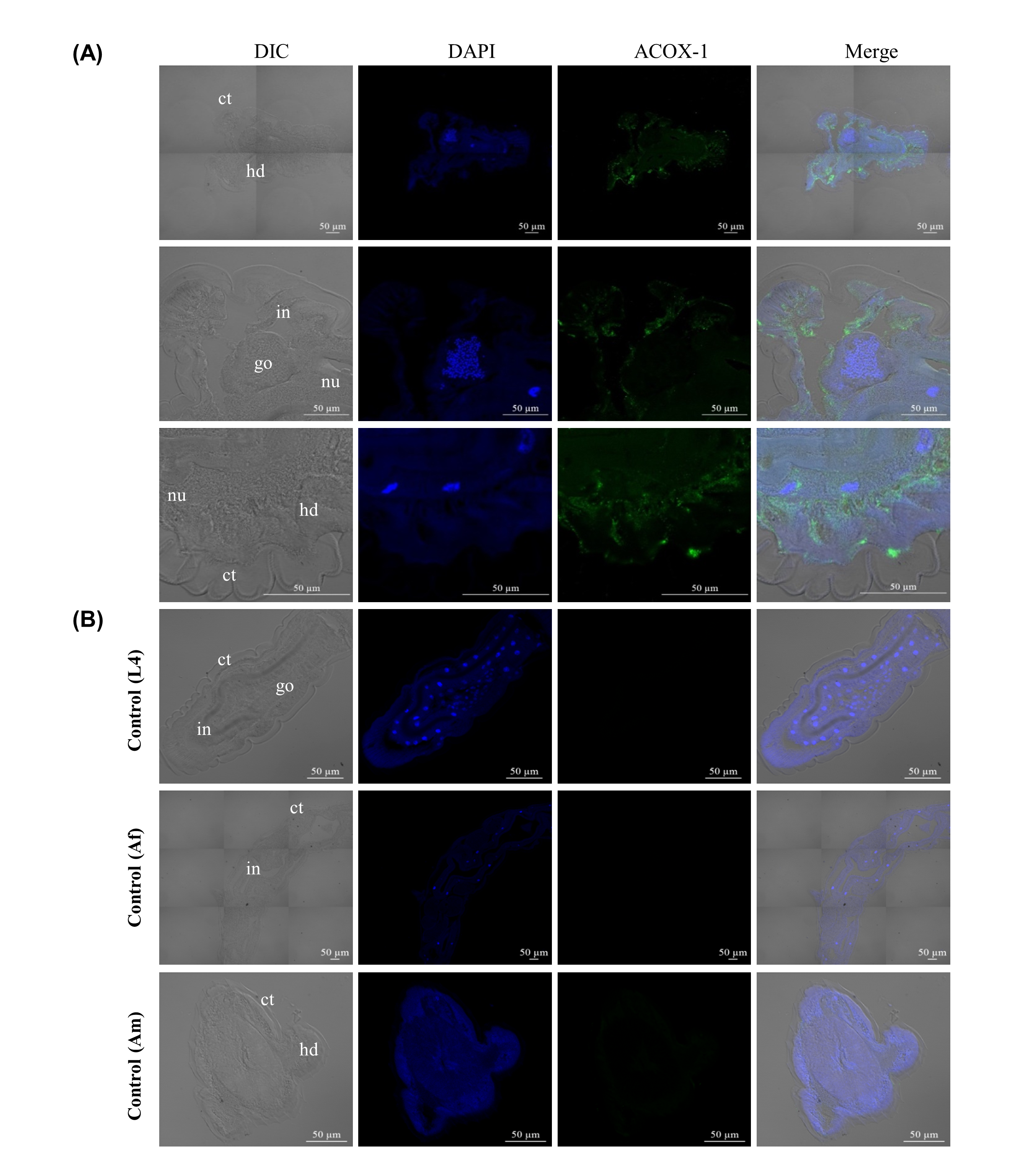

Supplement: S5 Fig — (A) Tissue distribution of Hc-ACOX-1 is indicated in the male adult worms of H. contortus, using anti-rHc-ACOX-1.1 polyclonal antibodies and DAPI. (B) Pre-immune serum is used as the primary antibody in negative controls for the fourth-stage larvae (L4s), female (Af) and male (Am) adult worms of H. contortus. Fluorescein conjugated goat anti-rabbit IgG (H+L) is used as the secondary antibody. DIC: differential interference contrast, ct: cuticle, in: intestine, go: gonad, nu: nucleus, hd: hypodermis. Scale bar: 50 μm. (TIF) [file ppat.1009767.s006.tif]
